# Zinc isotope variations in archeological human teeth (Lapa do Santo, Brazil) reveal dietary transitions in childhood and no contamination from gloves

**DOI:** 10.1371/journal.pone.0232379

**Published:** 2020-05-14

**Authors:** Klervia Jaouen, Manuel Trost, Nicolas Bourgon, Rozenn Colleter, Adeline Le Cabec, Thomas Tütken, Rodrigo Elias Oliveira, Marie Laure Pons, Pauline Méjean, Sven Steinbrenner, Jérôme Chmeleff, André Strauss

**Affiliations:** 1 Géosciences Environnement Toulouse, CNRS, Observatoire Midi Pyrénées, UMR 5563, Toulouse, France; 2 Department of Human Evolution, Max Planck Institute for Evolutionary Anthropology, Leipzig, Germany; 3 AG für Angewandte und Analytische Paläontologie, Johannes Gutenberg-Universität, Mainz, Germany; 4 Inrap, National Institute of Preventive Archeological Research, Cesson-Sévigné, France; 5 AMIS, CNRS, University of Toulouse UMR 5288, Toulouse, France; 6 PACEA, UMR 5199-CNRS, Université de Bordeaux, Pessac, France; 7 Laboratório de Arqueologia e Antropologia Ambiental e Evolutiva, Instituto de Biociências, Universidade de São Paulo, São Paulo, Brazil; 8 CEREGE, CNRS, Aix Marseille University, IRD, INRAE, Coll France, Aix en Provence, France; 9 Museu de Arqueologia e Etnologia, Universityof São Paulo, São Paulo, Brazil; Ecole Normale Supérieure de Lyon, FRANCE

## Abstract

Zinc (Zn) isotope ratios of dental enamel are a promising tracer for dietary reconstruction in archeology, but its use is still in its infancy. A recent study demonstrated a high risk of Zn contamination from nitrile, and latex gloves used during chemical sample preparation. Here we assess the potential impact of the use of such gloves during enamel sampling on the Zn isotope composition of teeth from a population of early Holocene hunter gatherers from Lapa do Santo, Lagoa Santa, Minas Gerais, Brazil. We first examined the amount of Zn and its isotopic composition released from the gloves used in this study by soaking them in weak nitric acid and water. We compared Zn isotope ratios obtained from teeth that were sampled wearing nitrile, latex or no gloves. Finally, we performed a linear mixed model (LMM) to investigate *post hoc* the relationship between the gloves used for sampling and the Zn isotope variability in dental enamel. We found that the gloves used in this study released a similar amount of Zn compared to previous work, but only in acidic solution. Zn isotope ratios of teeth and the LMM identified no sign of significant Zn coming from the gloves when teeth were handled for enamel sampling. We hypothesize that Zn in gloves is mostly released by contact with acids. We found that the main source of Zn isotope variability in the Lapa do Santo population was related to the developmental stage of the tooth tissues sampled. We report identical results for two individuals coming from a different archeological context. Tooth enamel formed *in utero* and/or during the two first years of life showed higher Zn isotope ratios than enamel formed after weaning. More work is required to systematically investigate if Zn isotopes can be used as a breastfeeding tracer.

## 1. Introduction

Geochemists performing ion exchange column chromatography for Zn isotopes (^66^Zn/^64^Zn expressed as δ^66^Zn value) are well aware of contamination issues. The first work published on Zn stable isotope abundances by Maréchal et al. [1] reports procedural Zn blanks of 50 ng. The zinc concentration used for isotopic analyses is typically 300 ng ml^-1^. The origin of Zn contamination has been observed in different clean laboratories and investigated in detail in 2017 by Garçon et al. [2]. The main source of contamination comes from gloves, especially those made of nitrile, neoprene and latex. Reagents (acids, resins, Milli-Q water) and laboratory facilities may also involve other potential sources of Zn contamination. It has been noted that other contaminants may directly be related to body hygiene or care products used by the experimenter, such as some types of shampoo, sunscreen, tooth paste or make-up [3–6]. The elevated content of Zn in blanks can become a substantial problem when working with non-primate mammal teeth, which generally contain low amounts of Zn [7]. Sample solutions of tooth enamel can contain Zn contents as low as 400 ng. Consequently, Zn isotope ratio analyses may become biased by substantial Zn contributions from glove contamination. Unfortunately, while the blanks assessed during ion exchange column chromatography can detect contamination in the clean lab, these blanks cannot account for any Zn contamination occurring during tooth handling, enamel sampling or during dilutions performed prior to Zn isotope analyses. As much as possible, the sampling procedure should not involve any direct contact between the sampled enamel and the gloves, as the experimenter should hold the tooth by the root–if formed and/or preserved–, and use tweezers to collect the chunks of enamel. However, this contact may happen, especially when the experimenter is not aware of the risk of contamination or for teeth without any formed or preserved roots.

This study aims to assess how the use of gloves affects Zn isotope measurements of dental enamel, and to propose safe sample handling practices. To do so, we performed a series of tests to assess the potential of Zn contamination from several types of gloves used during the preparation of enamel samples later used in Zn isotope analyses (TEST 1). We specifically tested types of gloves which were not mentioned in Garçon et al. [2] and which are routinely used at the Max Planck Institute for Evolutionary Anthropology, where most of the preparatory and analytical work on dental enamel has been performed. We also considered the contamination coming from the fingertips of the gloves—in comparison to the whole gloves—since they come into direct contact with the teeth if the experimenter used any gloves during sampling. In a second step (TEST 2), we analyzed teeth to assess if substantial variation could be attributed to potential contamination during sampling from the experimenter wearing the different types of gloves used in TEST 1 (nitrile, latex, vinyl and no gloves). To perform TEST 2, we used different types of teeth (deciduous and permanent, premolars and molars) coming from a pre-colonial population from Brazil, the early Holocene foragers of Lapa do Santo whose mobility and diet have been previously studied [8–10]. In a third test (TEST 3), we indirectly explored *post hoc* the contamination through nitrile and latex gloves used during the sampling of the Lapa do Santo teeth. We chose to use a linear mixed model (LMM) similar to the one used by Bourgon et al. [7] to explore the source of Zn isotopic variability in dental enamel. We included in the model, the amount of Zn loaded in acid onto the ion chromatographic column (abbreviated as “ALC”, which stands for Amount Loaded onto the Columns) as a predictor. This amount is different than the Zn concentration in the teeth (used as a predictor in the previous model, [7]) since the Zn ALC depends on the amount of dental enamel sampled, which can vary depending on the preservation, as well as the area of the tooth sampled. If the Zn ALC is small, the final δ^66^Zn value is more likely to be impacted by the δ^66^Zn of the gloves. The use of the LMM allows us to predict if other factors can explain the Zn isotope variability observed in the teeth, such as the tooth formation time or the Sr isotope composition–an indicator of the geographical origin of the humans from Lapa do Santo.

With these three tests, we confirm the high risk of Zn contamination coming from nitrile and latex gloves when working in the clean lab, but also reveal the absence of a detectable contamination when gloves are used during enamel sampling. We show that Zn isotope variations observed in the teeth of Lapa do Santo are due to dietary transitions: from the placental diet to breastfeeding as well as before and after weaning. To confirm this result, we undertook an additional experiment (see supplementary data for details). We systematically analyzed the teeth of an adult and of a child coming from a different archeological context, the early modern period Jacobins convent of Rennes, France. We then confirmed the trend observed in the Lapa do Santo population, which opens perspective for the use of Zn isotopes as a potential tracer of the weaning age. Finally, the *post hoc* test performed using the LMM does not detect any contamination–the Zn ALC is indeed not a predictor for the variance of the Zn isotope ratios–whereas the LMM confirms the crown formation time of the tooth (i.e., prenatally until ~2 years of age vs. after full weaning) as a main driver of Zn isotope variation in teeth.

## 2. Material and methods

### 2.1 Background information on cultural and dietary behavior of humans from Lapa do Santo

The teeth come from the Lapa do Santo archeological collection, a site from the Lagoa Santa region in east central Brazil. The permit IPHAN 01514.002697/2011-97 allows the use of the selected teeth for isotope studies. Lagoa Santa is well known to the academic community since the works of the Danish naturalist Peter Lund in first half of the 19^th^ century. Since then, hundreds of archeological sites were located in this region, recording ~12,500 years of non-continuous human occupation in caves and open-air sites [11]. For the Early- and Middle-Holocene occupations, lithic technology, zooarcheology, osteological markers and multi-isotopic analyses indicate groups of foragers with low mobility and a subsistence strategy focused on gathering plant foods and hunting small and mid-sized animals but no megafauna. A high frequency of caries indicative of elevated consumption of carbohydrates is observed among women but not men [12]. Lithics include small flakes and cores of quartz [13] that were often used to process non-cooked starch plants [14]. Artefacts such as projectile points and axe blades occurred only marginally. Rock art abounds, including the oldest securely dated evidence of rock art in South America (10.5 cal ky BP). Representations include animals, filiform anthropomorphs, geometric motifs, manioc’s tubers and semi-lunar axes. Similar styles are found over a large area of Brazil [15].

Of particular importance are the numerous well-preserved Early Holocene (10.6–9.4 cal ky BP; 95.4% interval) skeletons recovered from the region, in general, and from Lapa do Santo, in particular. The skeletal population from Lapa do Santo is comprised of ~50 individuals from both sexes and all ages. At Lapa do Santo funerary rituals include primary burials, reduction of the body followed by secondary burial and pits filled with disarticulated and fragmented bones of a single individual [9]. Ancient DNA extracted from skeletons from Lapa do Santo indicates they are entirely nested within past and present Native American genetic diversity [16]. Because of the tropical environment, very few individuals yield enough collagen to provide dietary information, but based on the few collagen δ^13^C and δ^15^N data and zooarcheology, it seems that the population relied on terrestrial resources with a small contribution of meat in their daily meals [9]. However, fish bones have been also found on the site [17], revealing a broad dietary spectrum.

### 2.2 TEST 1: Experiments on gloves

#### 2.2.1 Leaching experiments

**Fig 1:**
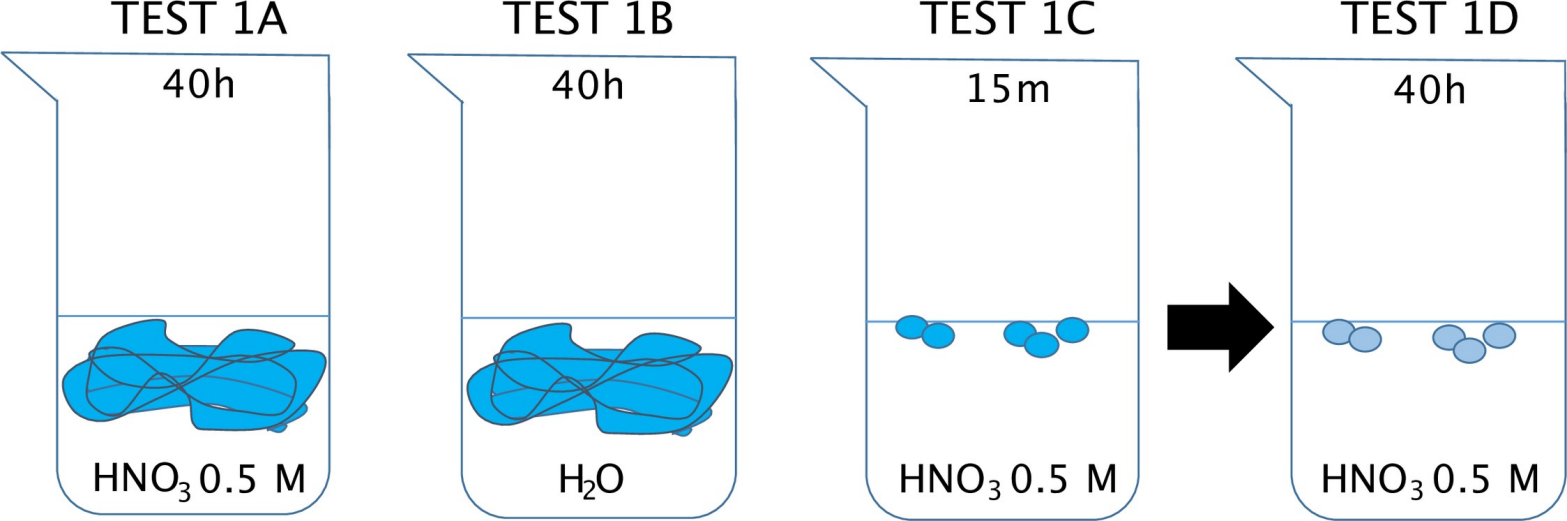
Schematic setup of the different leaching tests of gloves in acidic and aqueous solution. See text for more details.

Following the work by Garçon et al. [2], we selected gloves that are commonly used in our laboratories (Table 1) and that had not been tested yet as sources of Zn contamination. The experiments were conducted in the Pico Trace clean lab at the Department of Human Evolution, Max Planck Institute for Evolutionary Anthropology in Leipzig, Germany. The acids used in the experiments were ultrapure, certified to contain less than 100 ppt of Zn. The resin was rinsed 10 times with HNO_3_ 0.5 M, and the column steps included additional cleaning steps. All the lab consumables used for sample preparation and analyses were soaked 48 h in HCl 2 M. We used four different setups (Fig 1) to test the contamination issues. In order to avoid the sampling of their inner coating, the gloves were first tied with a knot (TESTS 1A and 1B). This, of course, does not apply to TESTS 1C and 1D, where the fingertips of the gloves were soaked. For TEST 1A, the complete gloves were soaked for 40 h in 100 ml of a solution of HNO_3_ 0.5 M in glass beakers (400 ml) first autoclaved and cleaned with Milli-Q water. The beakers were covered with parafilm and placed under a fume hood with laminar flow and extraction for the time of the experiment, to avoid any external contamination. Duplicates were run in 200 ml of solution, in 800 glass beakers. Then 10 ml of solution was sampled, placed in a Teflon beaker and dried overnight. The residue was then digested in 1.5 M HBr, and purified using the protocol described in Jaouen et al. [17] adapted from Moynier et al. [18]. Zinc isotope ratios and concentration were then directly measured on the Neptune MC-ICP-MS at the Max Planck Institute for Evolutionary Anthropology. For TEST 1B, a double distilled water solution was used instead of the HNO_3_ 0.5 M. For TEST 1C, the finger tips (a circle of about 1cm of diameter) of the gloves were cut using ceramic scissors and placed in beakers containing HNO_3_ 0.5 M for 15 min. For TEST 1D, the set-up is that of the TEST 1C but with a solution sampled after 40 h instead of 15 min. Ten ml of solution were first sampled, and a second sampling was performed 40 h later. The samples were then dried down on a hot plate, purified and analyzed following the protocol described for TEST 1A. To test the reproducibility of the results, TEST 1A was repeated for the nitrile gloves, 1B for the latex gloves (coated) and 1C for the nitrile and latex gloves (textured). For those duplicate analyses, the samples were prepared in the laboratories of Leipzig, but analyzed with a Neptune Plus at the Géosciences Environnement Toulouse department from the Observatoire Midi Pyrénées, France.

**Table 1:** List of the gloves tested in this study and associated characteristics. One glove per box was used.

Sample name	Type	Supplier	Model	Color	Size	Packaging	Reference number
N	Nitrile	ROTH	Rotiprotect nitril evo	Blue	M	Cardboard box	CPX7.1
LT	Latex textured	ROTH	Rotiprotect^®^-latex gloves Type 1 powder-free	White	M	Cardboard box	C269.1
LC	Latex coated	ROTH	Rotiprotect^®^-latex gloves Type 2 powder-free	White	M	Cardboard box	L.950.1
V	Vinyl	ROTH	Rotiprotect^®^-vinyl	Translucid	M	Cardboard box	6178.1

#### 2.2.2 Dry contact with tubes

**Fig 2:**
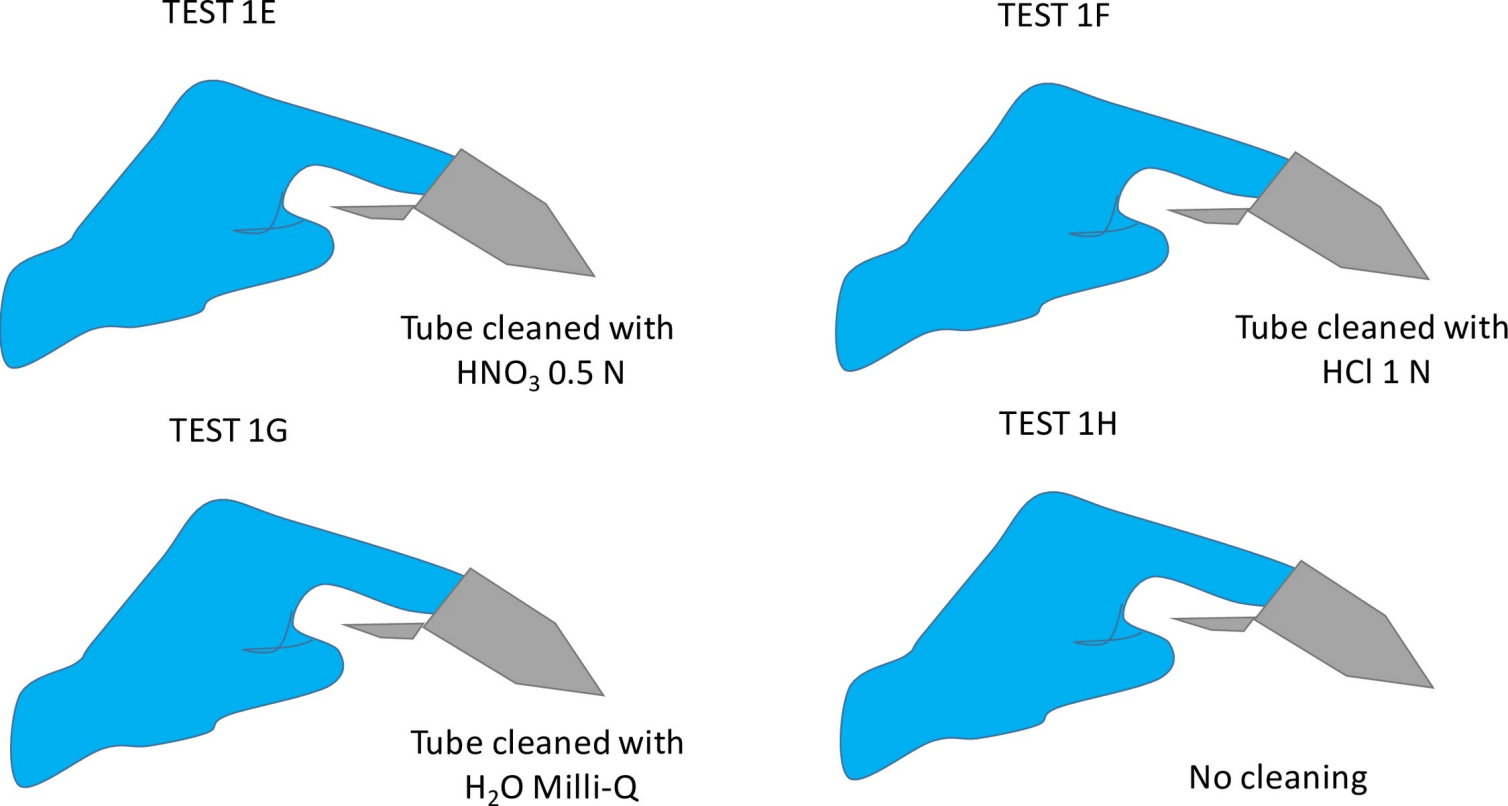
Schematic setup of the different dry contact tests with gloves and tubes cleaned in acidic and aqueous solutions. See text for more details.

Following again the example of Garçon et al. [2], we proceeded to a second glove contamination test in the context of dry contact with tubes. For this experiment, 5 ml-Eppendorf tubes were cleaned with HNO_3_ 0.5 N (TEST 1E), HCl 1 N–commonly used for labware cleaning–(TEST 1F), Milli-Q water (TEST 1G) or not cleaned at all (TEST 1H, Fig 2). The tubes cleaned with acids (TESTS E and F) were then soaked into water for one night, and all the tubes were dried under a fume hood. The experimenter then used the gloves listed in Table 1, introduced a finger for one second against the tube walls with a slight spin move, removed the finger and then added 1 ml of Milli-Q water. The tube was then shaken so that the liquid comes in contact with the whole surface of the tube walls, the solution is transferred to a clean Savillex, and the concentration was finally measured using the protocol described by Jaouen et al. [10].

### 2.3 TEST 2. Experiment sample contamination using different types of gloves

Twenty-six archeological human teeth (S3 Table) from 18 individuals of Lapa do Santo (Brazil) were sampled following our regular sampling technique using nitrile gloves (TEST 2A, n = 26), latex gloves (TEST 2B, n = 26) and no gloves at all (TEST 2C, n = 6). For this sampling technique, the enamel surface is first cleaned mechanically using a diamond-tipped burr before the chunk sampling (about 2 to 3 mm large and long). A microsaw is then used to sample a chunk of the tooth crown, from which the last traces of dentine are removed using a cleaned diamond-tipped burr, so as to only keep the enamel. All the teeth were sampled for TEST 2A and TEST 2B but unfortunately, only six teeth had enough material for further sampling for TEST 2C. The teeth were cleaned in Milli-Q water in glass beakers and put in an ultrasound bath for 15 min in order to remove the potential contaminants from the surface between each different test. In the case of TEST 2C, enamel powder was sampled rather than a chunk of tooth tissues to prevent the experimenters from injuring themselves while manipulating the microsaw on such a small object.

Prior to Zn isotope analyses, the zinc from the gloves and the teeth was extracted and purified using the protocol described by Jaouen et al. [18] adapted from Moynier et al. [19]. The isotope analyses were all conducted using a Neptune MC-ICP-MS at the Department of Human Evolution, Max Planck Institute for Evolutionary Anthropology in Leipzig, Germany. It is worth noting that for the teeth prepared with nitrile gloves, isotope compositions were analyzed at the LGPTE of the Ecole Normale Supérieure of Lyon, France, with a Nu-500 MC-ICP-MS, and duplicates were run using the Neptune Plus at the Géosciences Environnement Toulouse department from the Observatoire Midi Pyrénées, France. For each batch, the in-house standard AZE and the reference material SRM 1400 were run for interlab and interbatch comparison (S1 Table).

### 2.4 TEST 3. Post hoc assessment of contamination during sample preparation using linear mixed models

The linear mixed model (LMM) was performed using the statistical program R (version 3.6.1) [20]. To test the hypothetic predictors associated to Zn isotope variability, the model was fitted [21] with a Gaussian error structure and identity link [22] using the R-package “lme4” (version 1.1–17) [23]. The tested predictors were: the type of gloves (only nitrile and latex, samples obtained without gloves were excluded (n = 6) to ensure the model stability), ^87^Sr/^86^Sr isotope ratios-as a proxy for the geology-, the amount of Zn loaded on the column (ALC), the formation time of the tooth (group A: teeth formed before weaning, group B: teeth formed after weaning). All quantitative predictors were inspected for whether they were symmetrically distributed. They were also z-transformed to ease the model convergence (to a mean of zero and a standard deviation of one). In order to keep type I error rate at the nominal level of 0.05, a random slope of ALC was included [24,25]. We checked if residuals were normally distributed and homogeneous using a QQ plot (residuals plotted against fitted values) [26]. The model was tested on 52 teeth, coming from 18 individuals, and handled with two types of gloves.

## 3. Results

All the Zn isotope results and concentrations obtained in this study are available in the Supplementary Tables. The column Zn blanks for all the tests were found to range from 2 to 5 ng. The standards (in-house AZE and NIST SRM 1400) gave results consistent with the values previously published, but we noticed that the AZE samples prepared with nitrile gloves for the clean lab step gave lower results than the ones prepared with latex gloves (S2 Table, [18,27,28]). All samples but one showed mass-dependent fractionation for δ^66^Zn, δ^67^Zn and δ^68^Zn. The SRM 1400 standard gave consistent results in the three different laboratories where it has been measured with three different MC-ICPMS (Neptune, Department of Human Evolution/MPI-EVA, Leipzig; Nu 500 LGTPE/ ENS Lyon, Lyon; Neptune Plus, GET/OMP, Toulouse).

### 3.1 TEST 1: Experiments on gloves

**Fig 3:**
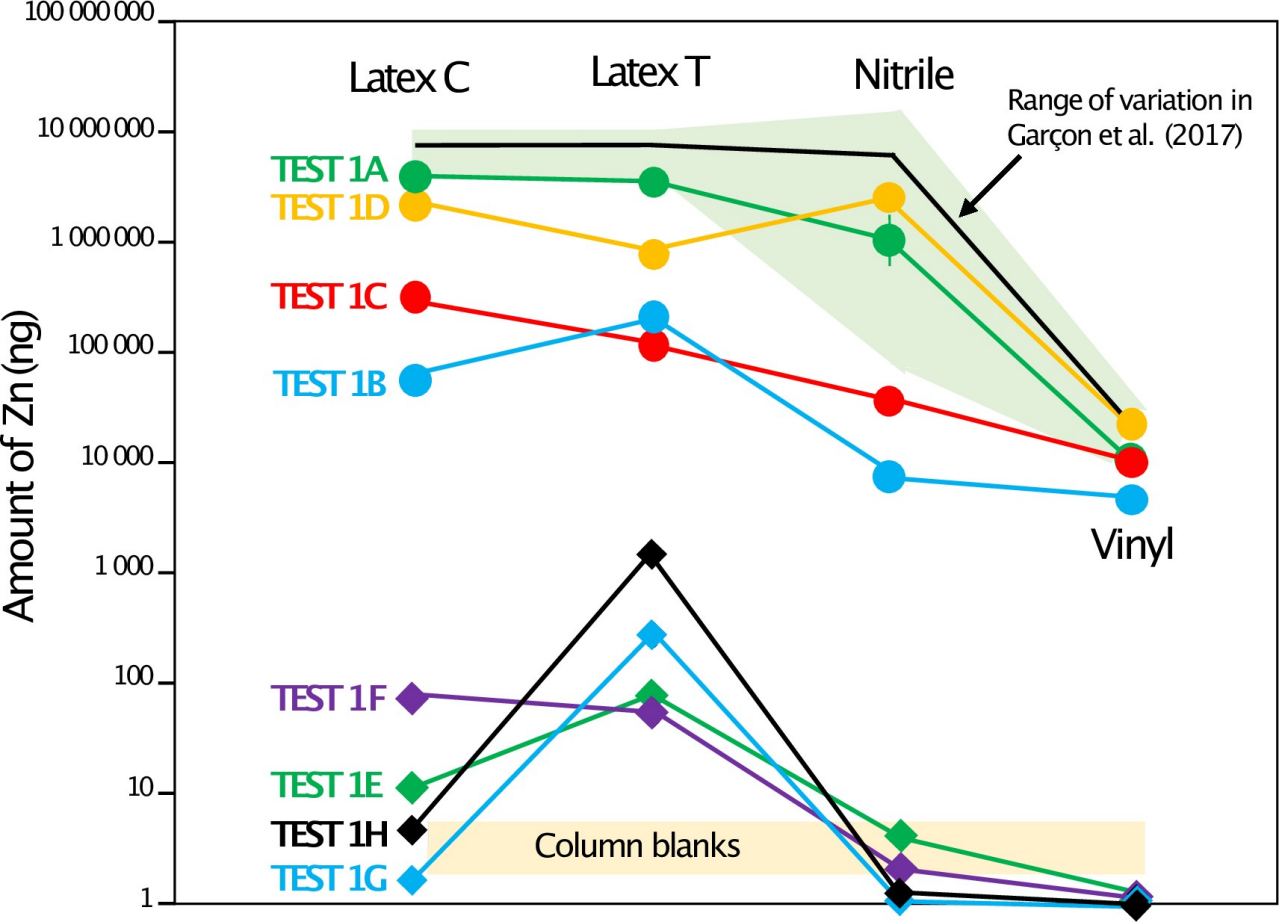
Amount of Zn obtained for each test and each type of gloves following the protocol described in Fig 1 and the corresponding text. “Latex C” corresponds to the coated latex gloves and “Latex T” to the textured latex gloves.

**Fig 4:**
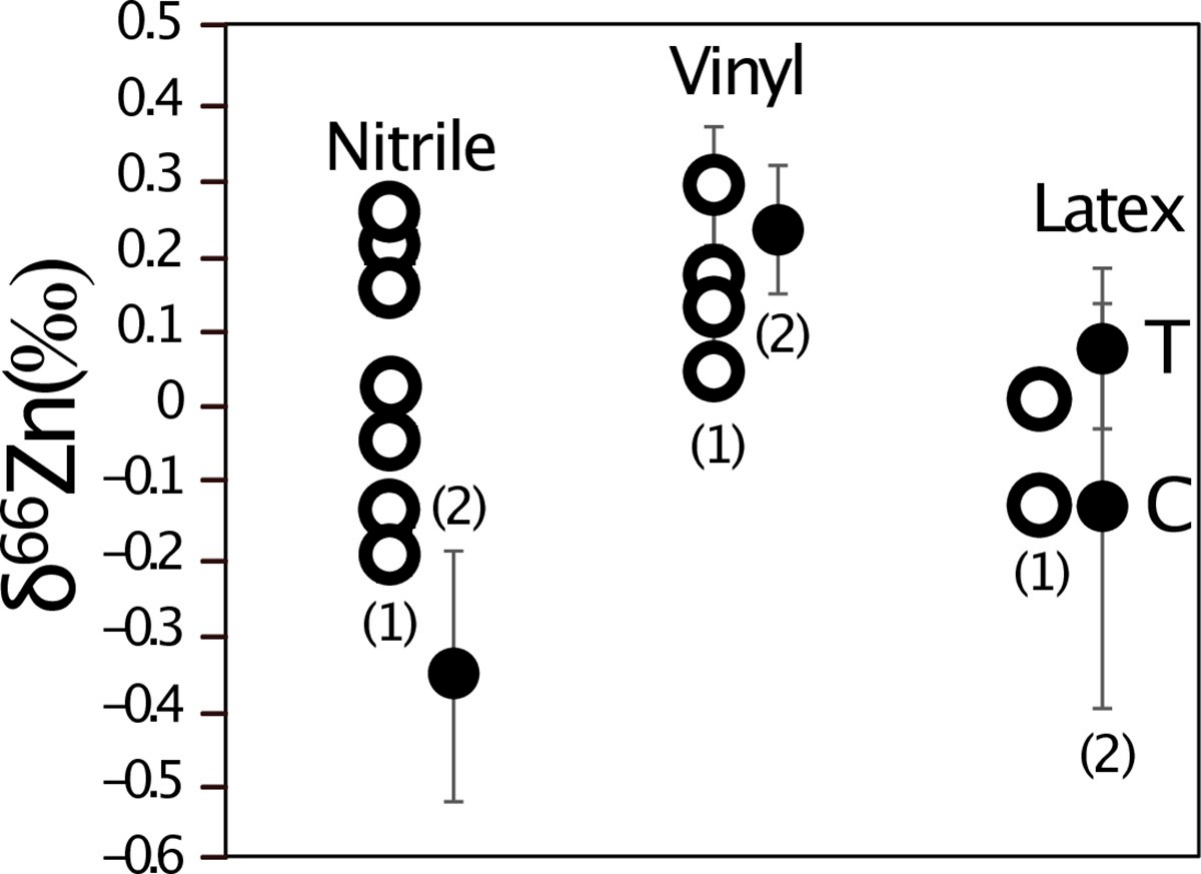
**Zinc isotope composition of the gloves from (1) Garçon et al. [2] (open circles) and (2) this study (filled circles).** For this study, the symbols represent the average value for the four tests and the whiskers correspond to the standard deviation. T stands for textured, C for coated.

**Fig 5:**
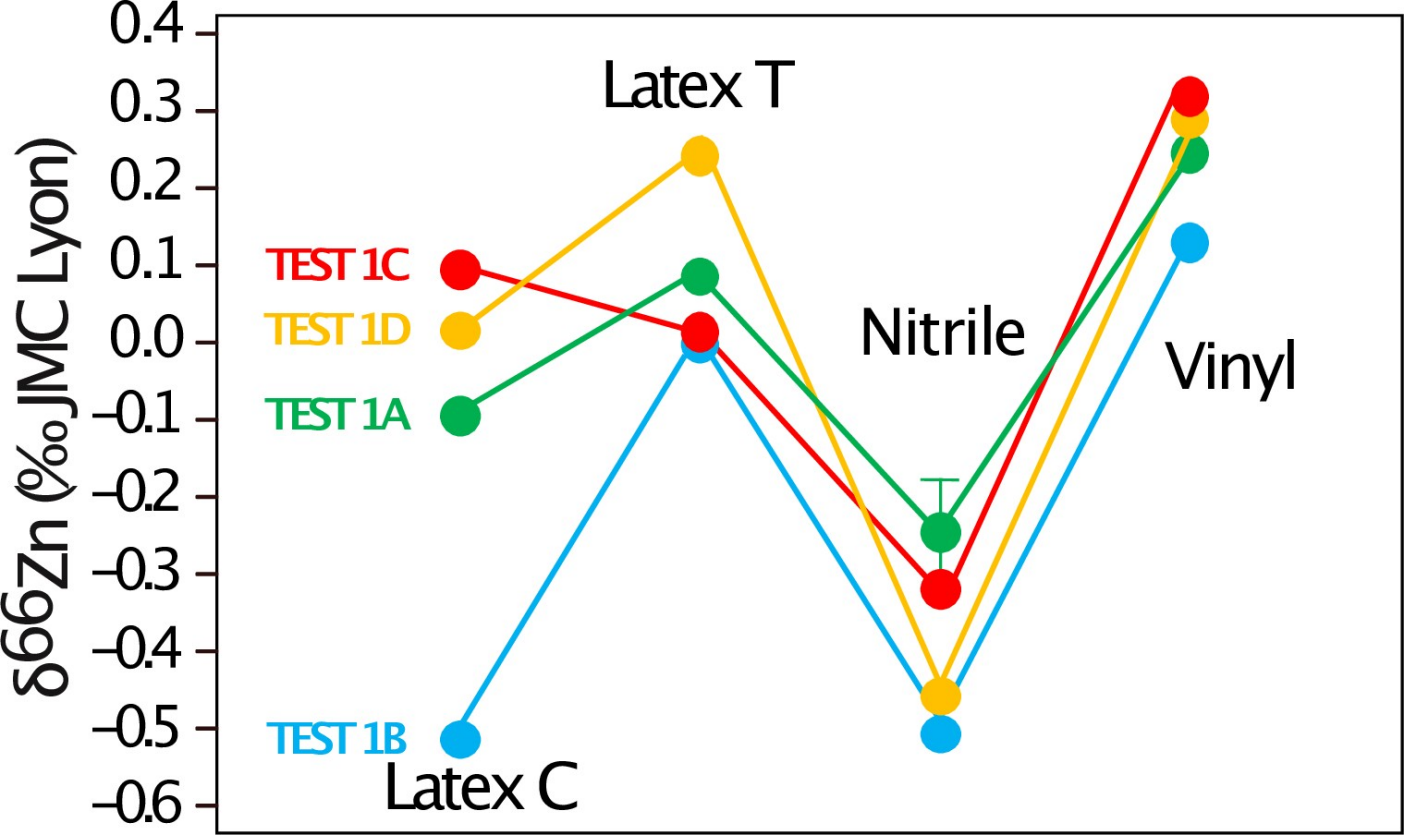
Zinc isotope ratios measured for the four types of gloves tested in this study. C stands for coated, T for textured. The description of the tests is provided in the text and Fig 1.

The amount of Zn released by the gloves following the tests by Garçon et al. [2] corresponds to the range reported by these authors (Nitrile and latex gloves release several grams, vinyl gloves about 50 mg, TEST 1A, Fig 3). Compared to the column blanks associated to the TEST 1 (2–3 ng), those amounts are enormous. The duplicates, ran in a different solution volume, released similar amounts of Zn (S2 Table). Because of the high matrix content of the samples, a column purification was performed prior to Zn isotope analyses of the glove test samples. The isotope ratios for the nitrile gloves were found to be the lowest values reported for any type of gloves (Fig 4). The isotope results were exactly the same for 3 of the 4 duplicates, the last one being shifted by 0.2‰ (Fig 5, S2 Table). The tests on the finger tips (TEST 1C and 1D) reveal that 14% and 50% of the Zn accumulated over 40 h is released respectively from the latex (coated or textured) and vinyl gloves in the first 15 min, while only 1% of the total amount of Zn recovered in 40 h from the nitrile gloves is released during this period. The amount of Zn released by the fingertips (TEST 1D) of the nitrile and latex gloves was comparable to the amount released by the whole gloves (TEST 1A, Fig 1). The latex gloves released the same amount in TEST 1A, while the fingertip tests (TESTS 1C and 1D) showed slightly higher amounts for the gloves with inner coating. This can be explained by a higher Zn content of this coating in comparison to the textured gloves, since the coating was only exposed to the solution in TESTS 1C and 1D. TEST 1B shows that a small portion of Zn is released by the gloves when they are soaked into water (more than two orders of magnitude lower for the latex gloves). As mentioned above, the column blanks are 2 to 3 ng. The blanks coming from the solutions in the glass beakers without gloves that were sampled (10 ml), evaporated, and purified on the columns like the actual samples (Fig 1) were also 3 ng, with the exception of two of them (100 ng). We assume that the contamination comes from the glass beakers, even though they were rinsed with Milli-Q water. The beakers were stored outside of the clean lab, used by experimenters working with nitrile gloves, and were possibly cleaned at different instances using an autoclave. The δ^66^Zn values of the different tests show substantial variation, but there is no correlation between concentration and isotope ratios. There is also no systematic difference between the ratios measured in the TESTS 1D and 1C, which argues for the absence of kinetic Zn isotope fractionation. We noticed however, that the δ^66^Zn values observed in TEST 1B are systematically lower than in the other tests (Fig 5).

TESTS 1E, 1F, 1H and 1G show similar amounts of Zn released from the gloves than those of the TEST B from Garçon et al. [2], except for the latex textured gloves that we tested which release substantially more Zn during a one-second dry contact. Surprisingly, those gloves also released more Zn when the tubes were not cleaned with acids, in contrast to all the other gloves which had the highest content in the tubes formerly cleaned with HNO_3_ and HCl.

### 3.2 TEST 2: Enamel sampling of teeth with different types of gloves

**Fig 6:**
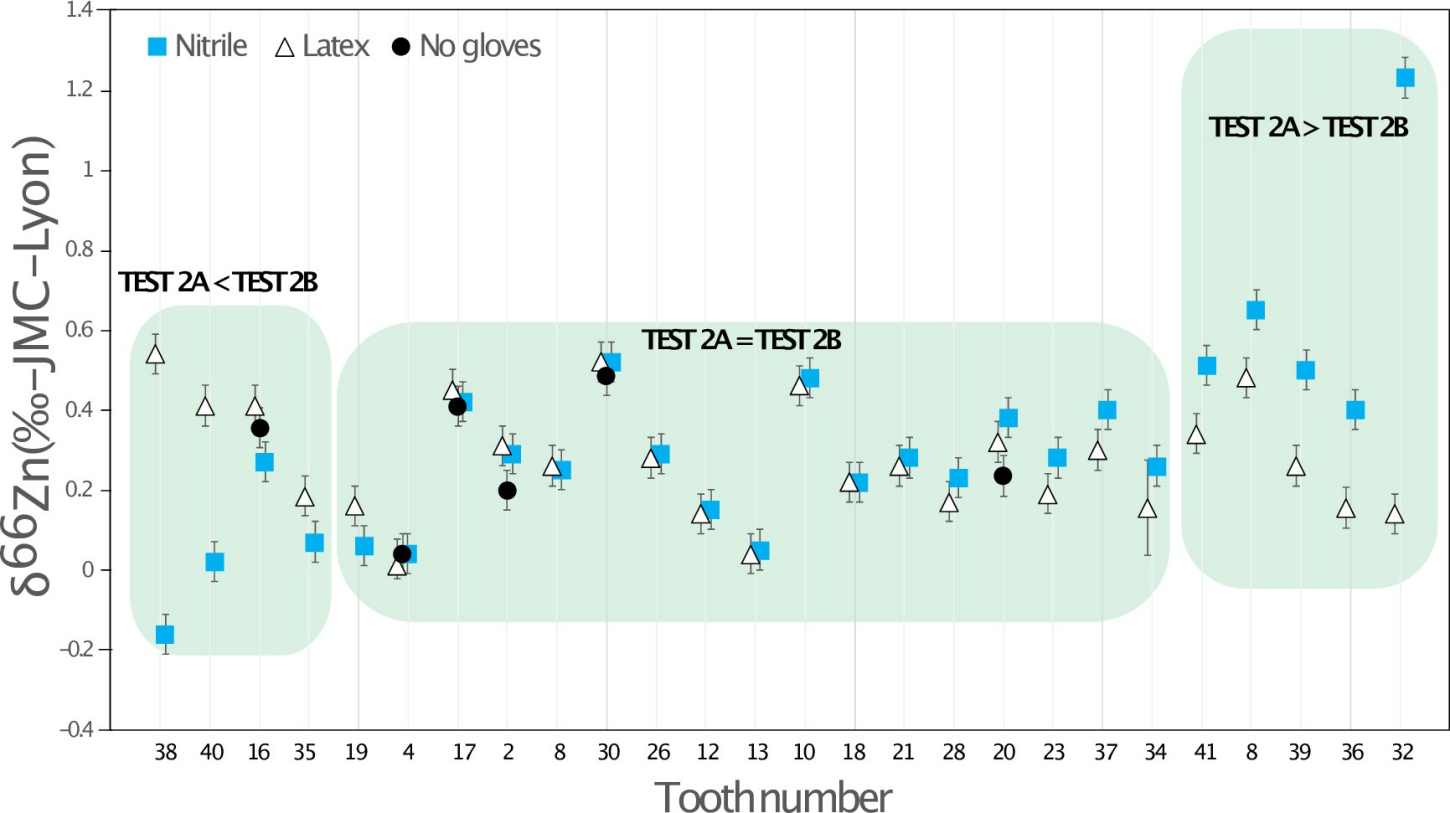
Zn isotope ratios for each Lapa do Santo tooth sampled either with nitrile gloves (TEST 2A), latex gloves (TEST 2B) or without gloves (TEST 2C). The numbers on the x-axis correspond to the tooth specimen ID (see S3 Table), to note that several teeth may belong to the same individual. Typical analytical error for Zn isotope ratios is 0.05‰ to 0.08‰ (2 SD), and the SD of the different standards (AZE and SRM 1400) run on the columns ranges from 0.08 to 0.1‰ (2 SD).

**Fig 7:**
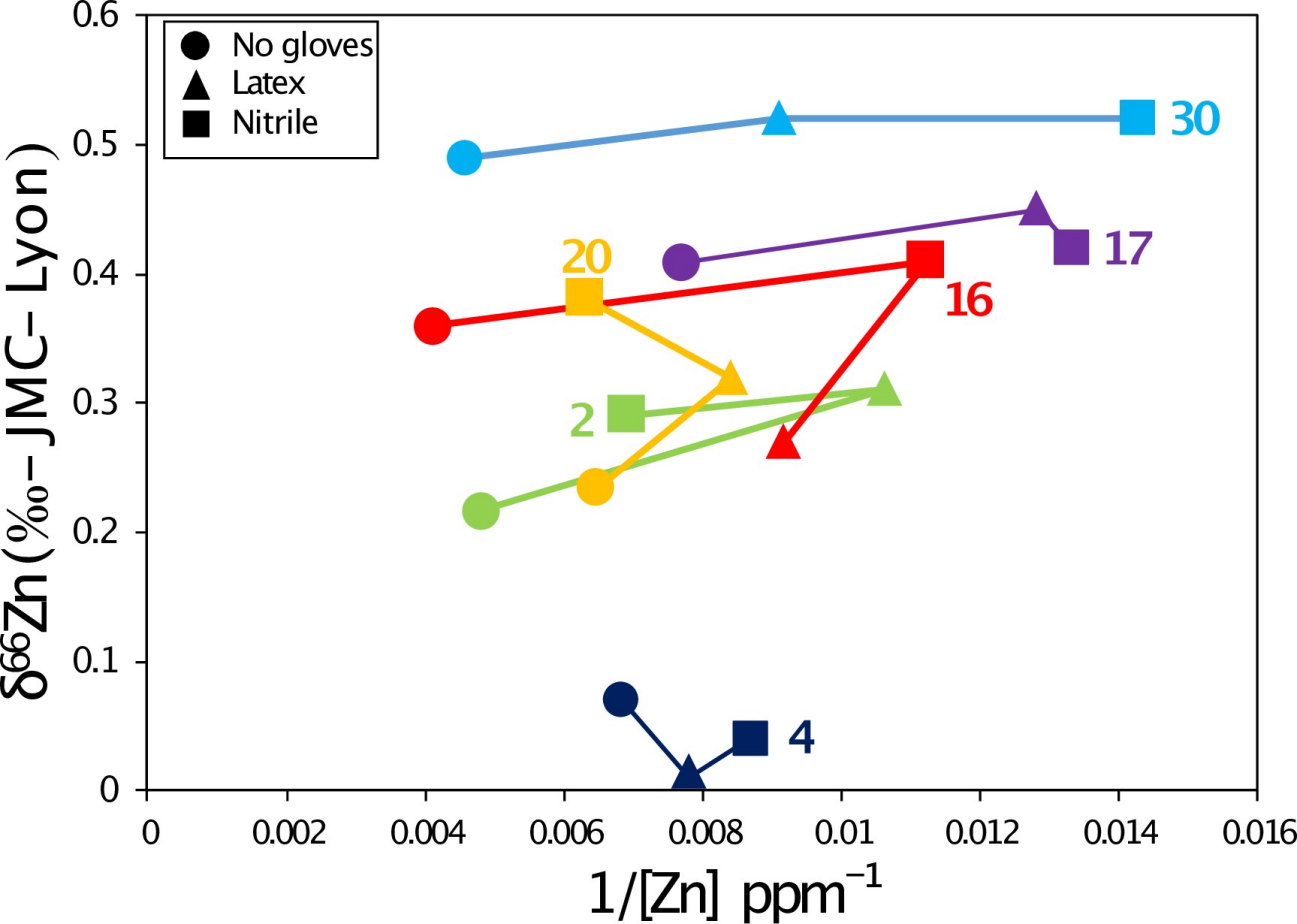
Relationship between Zn concentrations and isotope values for the six teeth on which all three TESTS 2 A, B and C have been performed. The numbers 2 to 30 corresponds to the identification number of the teeth, which are also given in the Supplementary Tables.

For the 26 Lapa do Santo teeth sampled, 62% gave identical results in TESTS 2A and 2B. For the remaining 10 teeth, four showed higher Zn isotope ratio values when sampled using latex gloves, and six of them higher values when sampled with nitrile gloves (Fig 6). TEST 2C only consists of six teeth sampled without using gloves. Among them, three showed values identical to those sampled with nitrile and latex gloves (Figs 6 and 7), and two had slightly lower values (0.1 to 0.2‰) than those sampled with latex and nitrile gloves, which were identical. The last sampled tooth showed a higher result in TEST 2B than in TEST 2A, the value in TEST 2C was similar to that in TEST 2B.

**Fig 8:**
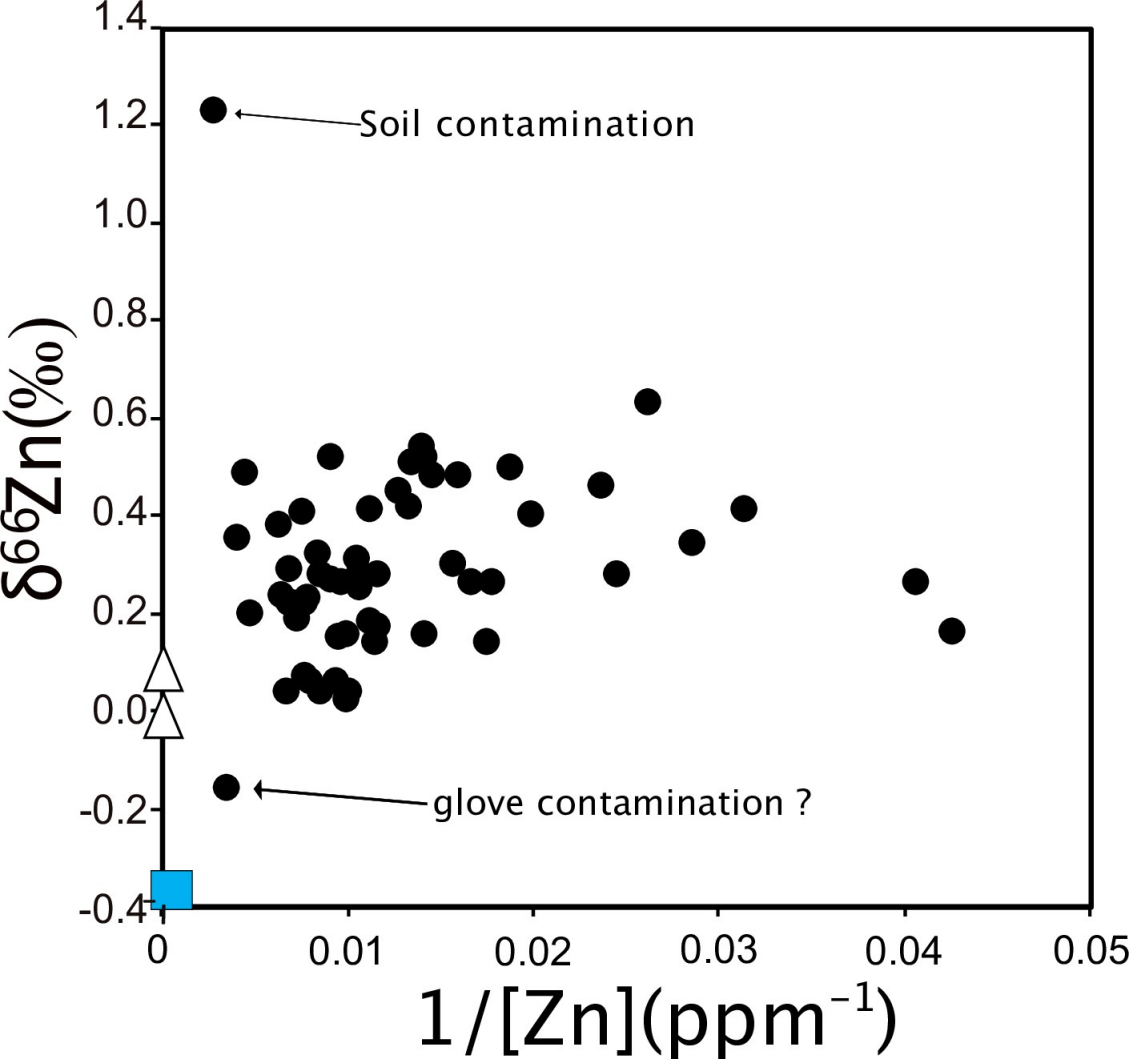
Relationship between Zn concentrations and δ^66^Zn values for the Lapa do Santo teeth (black circles). All samples are plotted together, independently from the sampling method used. The blue square represents the nitrile gloves, and the empty triangles the latex gloves.

**Fig 9:**
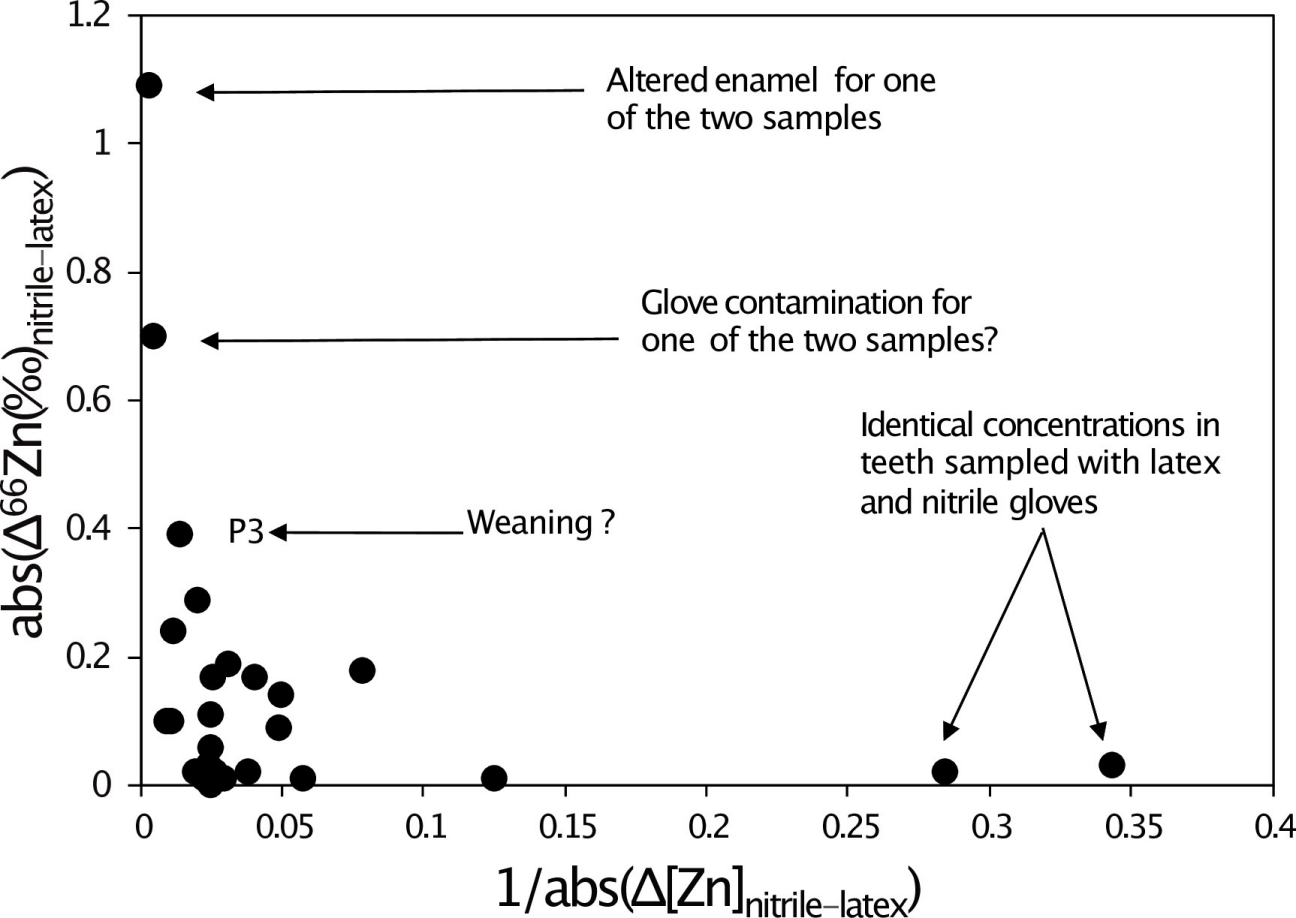
Correlation between the difference of concentration and the difference of δ^66^Zn values of tooth samples from Lapa do Santo prepared with nitrile or latex gloves. P3 = first premolar. The typical analytical error for Zn isotope ratios ranges from 0.05 to 0.08‰ (2 SD), and the SD of the different standards (AZE and SRM 1400) run on the columns are 0.08 to 0.1‰ (2 SD).

We observe no correlation between Zn concentrations and isotope ratios (Fig 8). This was also observed for the additional teeth from the Jacobins convent (Rennes, France), which are described in the supplementary information. There are two outliers, one with a very positive δ^66^Zn value and another one with a low δ^66^Zn, which was sampled with nitrile gloves. The latter one is also the only sample showing mass-independent fractionation (see arrows on Fig 8), which is generally caused by isobaric interferences. This can happen when too much matrix is present in the solution knowing that the gloves release many other elements than just Zn [2]. The two outliers also have drastically different δ^66^Zn values and Zn concentrations compared to the two samples extracted from the same teeth and prepared with nitrile and latex gloves (Fig 9).

In Lapa do Santo, no differences in δ^66^Zn were observed between individuals identified as men or women (Kruskal-Wallis χ^2^ = 0.3229, df = 1, p-value = 0.57). The C, N and Sr isotope data also did not reveal differences between sexes [8,9]. Interestingly, the type of tooth is strongly associated to specific δ^66^Zn values (Kruskal-Wallis χ^2^ = 28.32, df = 6, p-value = 0.000082, all measurements taken together; n = 77 and Kruskal-Wallis χ^2^ = 14.98, df = 6, p-value = 0.02, average value per tooth, n = 26). This trend is even more significant (Kruskal-Wallis χ^2^ = 27.35, df = 3, p-value = 0.0000050, all measurements together, n = 77) when the teeth are grouped by formation periods, which are themselves related to dietary transitions in childhood (deciduous molars: placental and exclusive breastfeeding; permanent first molars (M1): breastfeeding, with and without solid food; permanent first premolars (P3): weaning and post-weaning diet; second premolars, permanent second and third molars (P4, M2, M3): post-weaning diet).

### 3.3 TEST 3: Linear mixed model

The full-null Linear Mixed Model comparison was significant (likelihood ratio test: *Χ*^2^ = 10.62, df = 8, p-value = 4,95 10^−3^) and allowed to assess which of the tested predictors were associated with variations in δ^66^Zn values. The tooth formation time (i.e., *in utero* and before weaning vs. post-weaning) and the Sr isotope ratios showed a significant relation with δ^66^Zn values (likelihood ratio test p-values equal to 0.002 and 0.039, respectively). The ALC and the type of gloves used for sampling appeared to have no significant relation with the variability of the δ^66^Zn values (likelihood ratio test p-values equal to 0.158 and 0.482, respectively).

## 4. Discussion

### 4.1 Zinc contamination during sample preparation in the clean lab

Our study confirms the strong risk of Zn contamination from nitrile and latex gloves, especially when working with acids. Vinyl gloves release a smaller amount of Zn (two orders of magnitude) during acid exposure than the two other types of gloves (nitrile and latex). Nitrile gloves release Zn that is depleted in heavy isotopes compared to the other brands of nitrile gloves studied by Garçon et al. [2] (Fig 4). The four types of gloves release similar amounts of Zn in comparison to the study of Garcon et al. [2] (Fig 3). This suggests that the conclusions of the two other experiments (TEST 2 and TEST 3) conducted on different types of gloves of the Rotiprotect brand can be of interest for users of other brands. We recommend that when working with acids and wearing gloves made of latex, nitrile or neoprene, the experimenters should wear a second pair of gloves on top made of vinyl to lower the risk of Zn contamination. Some laboratories also adopt the strategy to wash the gloves with water prior to Zn purification in the laboratory. However, our tests show that a limited amount of Zn is released in water. As mentioned by Garçon et al. [2], the best strategy is to avoid any contact between the gloves and the cleaning solutions for the beakers, the pipette tips or any labware that comes in contact with the samples. This strategy has proved to be efficient in our clean lab where the blanks are usually around 2 ng and never exceeded 10 ng, since we have become aware about the contamination risk by gloves. We also noticed a small change of the δ^66^Zn value of our in-house Zn isotope standard and an improvement of the standard deviation around this value (S1 Table). This could be related to the reduction of potentially glove-derived Zn contamination (now measured around 1.59‰ instead of 1.51‰, see [7] and [28]). We estimated that a contamination of 50 ng in the clean lab and 10 ng during the sample dilution prior to Zn isotope analyses would trigger a shift of about -0.1‰ for this in-house standard. We observed consistent values between the two above mentioned publications for the SRM 1400 (δ^66^Zn = 1‰, S1 Table) but this standard contains more Zn (181 ppm) than the in-house AZE (about 140 ppm).

### 4.2. Zinc contamination during the enamel sampling procedure

**Fig 10:**
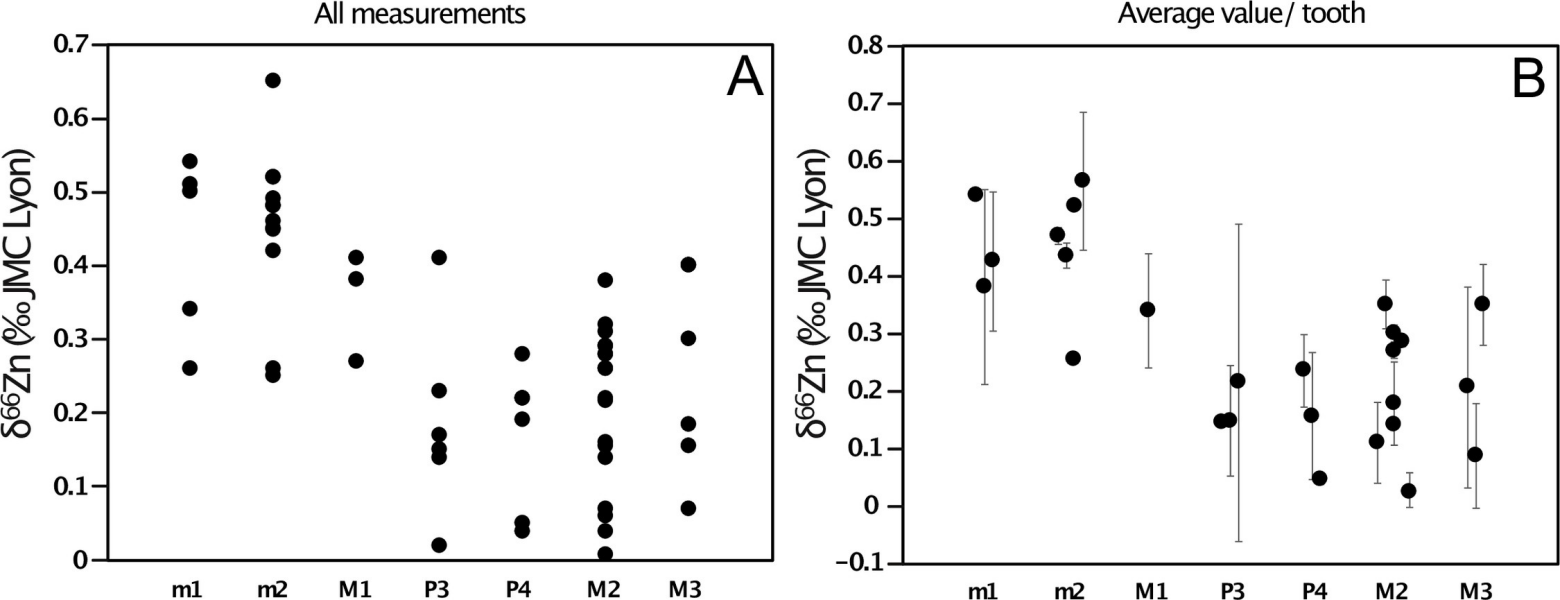
Zn isotope values in teeth of the Lapa do Santo population. Tooth types are ordered from the earliest to the latest crown initiation and formation times: from *in utero* (deciduous molars) to puberty (permanent third molar). A) All single measurements pooled together (sample preparation with gloves–nitrile or latex- or without), B) average δ^66^Zn value per dental specimen. The error bars represent the variation within a tooth (1 SD).

Most of the teeth sampled with two different types of gloves showed identical δ^66^Zn values in the analyzed enamel samples, whereas nitrile and latex gloves used for these experiments had very different δ^66^Zn values. The multiple samples taken from a single tooth do not represent a homogenized powder, but were rather taken from different parts of the tooth crown, thus corresponding to different times of enamel formation and thus potentially reflecting differences in diet-related δ^66^Zn values. For the tooth samples showing different δ^66^Zn values, no systematic trend towards more positive or negative values could be observed using one or another type of gloves or no gloves at all (Fig 6). The LMM results confirm the lack of influence of the glove type used for sampling on the Zn isotopic variability. This could result from the release of Zn mostly occurring in an acidic environment (see results from the TESTS 1 A to H). It appears that the teeth showing the maximum variation in δ^66^Zn values, form their enamel during major periods of dietary transition in human life history, which are birth and weaning (Fig 10B). The LMM also demonstrated that the δ^66^Zn values are linked to the formation time of the tooth. Diet is usually known to be the main source of Zn isotope variations in human teeth [18,27,28]. Strontium isotope ratios are related to the bioavailable Sr from the local bedrock substrate and therefore reflect the geographical origin of the studied individuals. In Lapa do Santo, these Sr isotope ratios were also found to correlate with δ^66^Zn values, which was already highlighted for well-preserved fossil mammal teeth by Bourgon et al. [7] using a similar LMM. This means that, although Zn is mainly influenced by diet, the geology of the area where the food products come from can also influence, to a certain degree, the Zn isotope ratios recorded in teeth. This is expected since marine limestones and some siliceous sediments show much higher δ^66^Zn than other types of rocks [29]. Although both studies revealed the relationship between Sr and Zn isotope ratios, a potential bias in the Lapa do Santo teeth relates to the fact that the previously published Sr isotope ratios [9] were obtained from teeth that may differ from those used in the present study. When multiple samples coming from different teeth or within a tooth of the same individuals were analyzed for Zn, a unique Sr isotope ratio was associated. Even if a potential bias exists for the relationship between Sr and Zn isotope data, the variation observed between the two or three sampling tests (Fig 7) is therefore biogenic and not due to contamination, with one possible exception (Fig 6). Contamination related Zn isotopic shifts are often accompanied by a change of Zn concentration (Fig 8).

In summary, the teeth showing the largest isotopic variations may involve 1) a soil contamination (δ^66^Zn = 1.23‰, Figs 6, 8 and 9); 2) an analytical problem or a glove contamination resulting in samples showing mass-independent fractionation (δ^66^Zn = -0.16‰, Figs 8 and 9); 3) enamel forming during dietary transition periods. For this latter category, the maximal variation did not exceed 0.4‰, which may reflect a dietary shift by one trophic level [7,10,26].

### 4.3 Life history and dietary effects on human enamel δ^66^Zn values

The Zn isotope composition of archeological teeth, when not impacted by glove contamination during sampling, is strongly related to the developmental times at which enamel starts forming. Little is known about Zn metabolism and absorption mechanisms during lifetime dietary transitions. Zinc absorption is not solely related to Zn intake [30]. Phytates, contained in plants–- especially in cereals—constitute inhibitors to Zn absorption. However, infants whose mothers feed on a cereal-based diet show higher Zn/Ca ratios in their prenatal enamel (i.e., formed *in utero*) than other infants [30]. This can be explained by the fact that during pregnancy, women having the lowest daily Zn intake also have the highest rate of Zn absorption [31]. This regulation stops during lactation. In another study, Donangelo et al. [32] not only confirmed that the types of diet with high amount of phytates were correlated with higher Zn absorption rates during pregnancy, but they also observed the effect during lactation. Zn absorption among pregnant women remains generally higher than in non-pregnant and non-lactating women [33,34]. Interestingly, however, Zn/Ca measured in modern populations from Mexico have been shown to be higher and more variable in enamel formed postnatally than prenatally [2,35]. Infants that are breastfed show a Zn absorption around 41% [36] while in pregnant women it does not exceed 30% [31], which might explain this observed difference. Zinc concentration has also been shown not to be a reliable trophic level indicator [30] as opposed to Zn isotope ratios [7,18,28]. Prior to the present study, no work has addressed the question of Zn isotope fractionation in enamel formed *in utero* and during breastfeeding.

For the Lapa do Santo site, we had access to seven tooth types. Two of them—deciduous first and second molars (m1 and m2, respectively)—had crowns partially formed *in utero* and during the first months of life (Fig 10, [37]). The permanent first molars (M1) form their crown during the first three years of life, and therefore likely record the isotope signature associated not only with breastfeeding, but also with the introduction of solid food in the infant’s diet. The crown of the first premolar (P3) initiates around 1.5 years of age, when most hunter-gatherer infants still breastfeed [38–41] although solid food has already largely been introduced in their diet. Zinc absorption in infants feeding on mother milk is also much higher (around 40%) than that of individuals with omnivorous diet (around 24%, [36,42]) because Zn absorption is inhibited by phytates contained in plants, notably in high amounts in cereals [43]. Therefore, the isotope signature of Zn might be strongly influenced by the signature of human milk even after the introduction of solid food. The second premolar (P4) crown starts forming two years after birth [37] therefore recording a diet exclusively or mainly based on solid food. The second and third permanent molars (M2 and M3) are formed after weaning. When teeth are ranked depending on their time of initiation and formation, a trend in enamel δ^66^Zn values can be seen, with the highest values in teeth initiating their enamel formation *in utero* and the lowest values in teeth formed after the weaning age (Fig 10).

**Fig 11:**
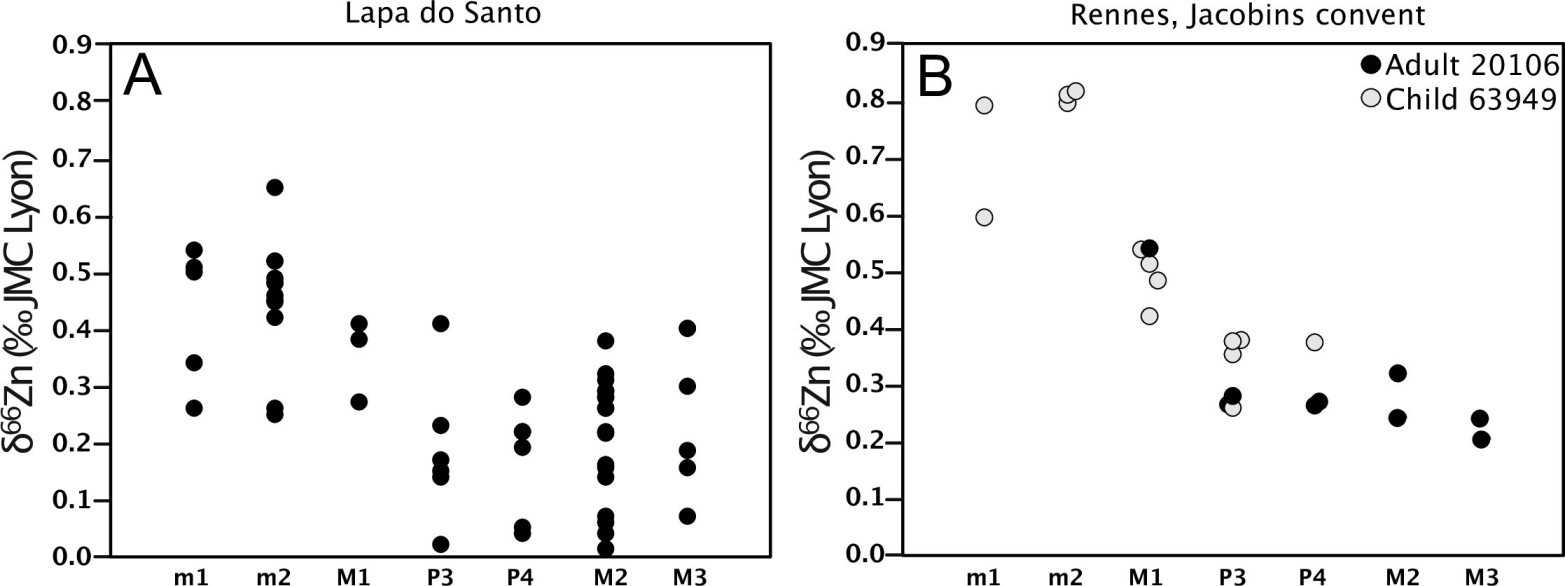
Zinc isotope values from enamel of different tooth types at (A) Lapa do Santo (Brazil), and (B) Jacobins convent (Rennes, France). Note the trend of decreasing δ^66^Zn values in enamel with increasing crown formation age for humans from both archeological sites.

**Fig 12:**
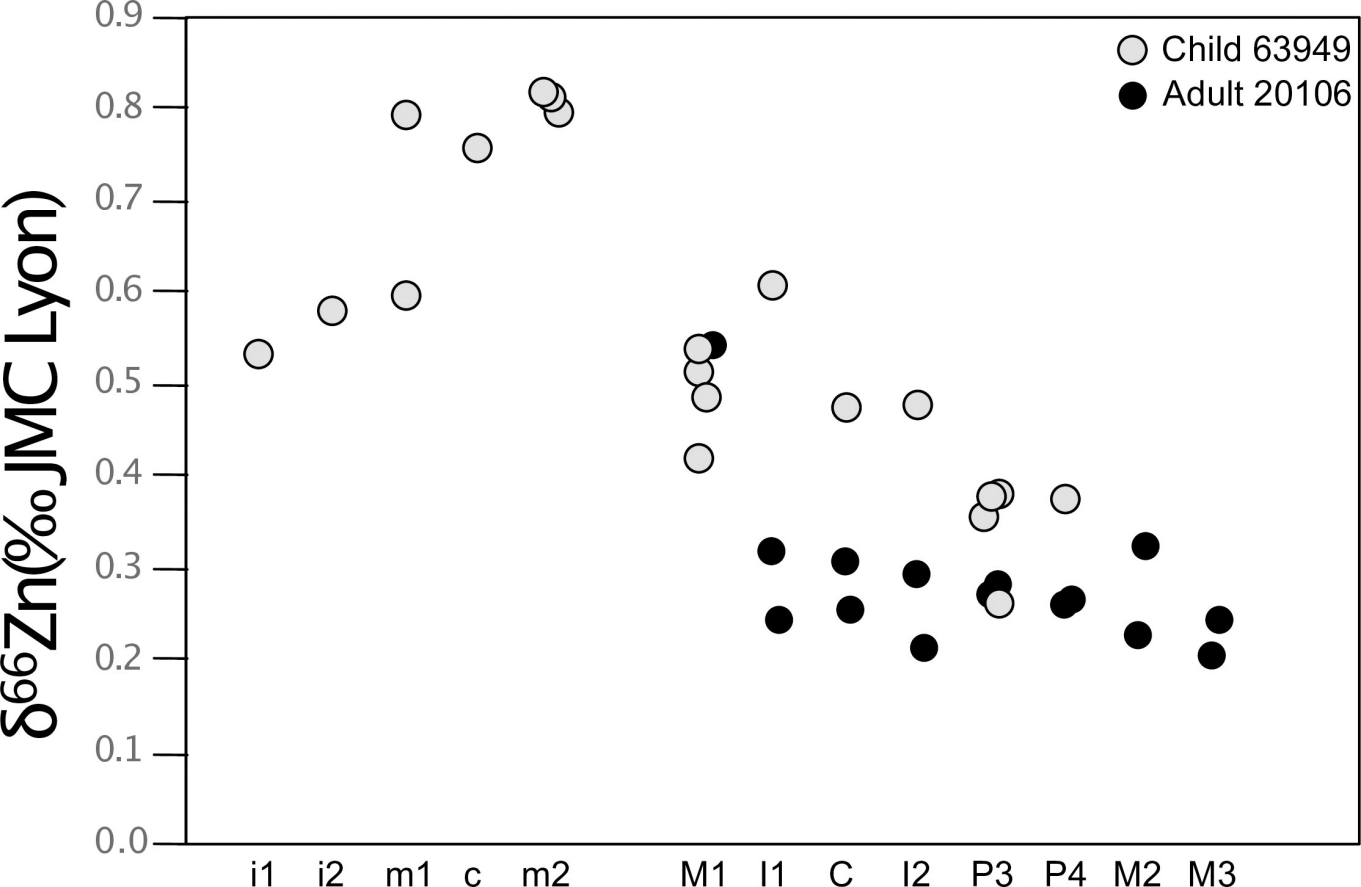
Zinc isotope compositions of all the tooth types sampled for two individuals of the Jacobins convent. The teeth are ranked from the earliest forming enamel to the latest forming crowns.

In order to test the robustness of this ontogenetic trend, we decided to systematically analyze each tooth type from archeological remains of a child (estimated age at death: 5–9 years, see Supplementary Information) and an adult. As it was not possible on the Lapa do Santo material, this was undertaken on two specimens belonging to a medieval population recovered at the Jacobin convent, in Rennes, France. The diet of these individuals has already been extensively documented [27,44]. The procedure of this test is fully described in the Supplementary Information. Every tooth type analyzed from the two Les Jacobins individuals yielded a pattern very similar to that observed at the scale of the Lapa do Santo population (Fig 11). This test especially confirms that the teeth starting to form their crown around birth have the highest enamel Zn isotope ratios, while the post-weaning teeth show the lowest values. As expected, the M1 have intermediate isotope signatures, as its crown formation time ranges from *in utero* until ~3 years of age. The teeth sampled in Rennes allow to clearly confirm the trend observed (Figs 11 and 12).

**Fig 13:**
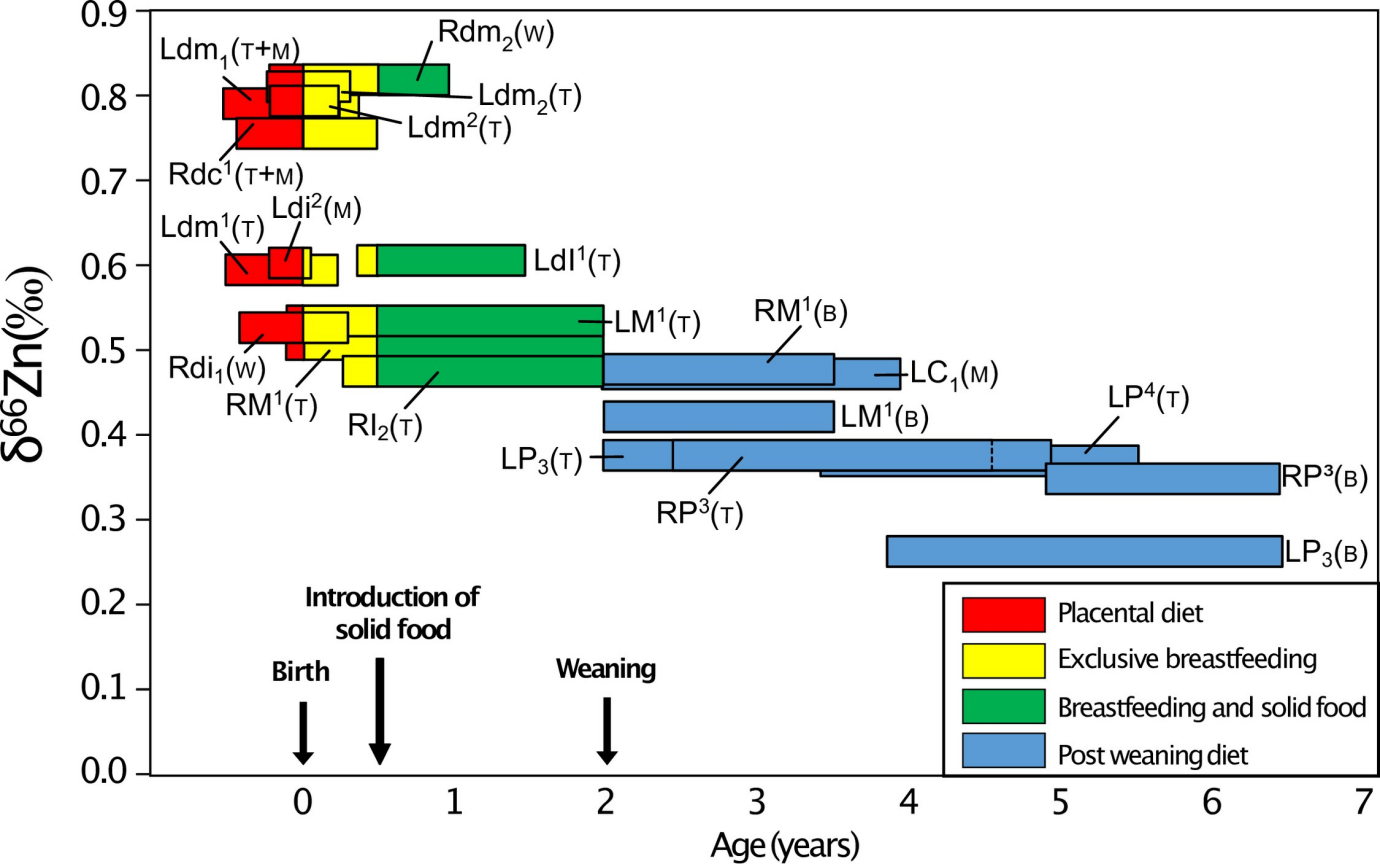
Zn isotope ratios in the different teeth of Child 63949, recovered from the Jacobin convent (Rennes, France), with the corresponding formation ages of the areas of enamel sampled (S5 Table) [37]. Different parts of the tooth crowns were sampled: top (T); middle (M); bottom (B); whole height (W). The age at introduction of solid food and weaning is here fully hypothetical (i.e., there is no archeological record for this specimen) but is based on nitrogen isotope data measured in the tooth roots as well as historical data on the population (Supplementary Information). Because the actual weaning age could be slightly younger or older, it is provided only for illustrative purposes.

For the Jacobin convent, we carefully documented the location of the sampling on the teeth, which allowed estimating an age of enamel formation for each sample (S5 Table, S2 Fig, Fig 13).

**Fig 14:**
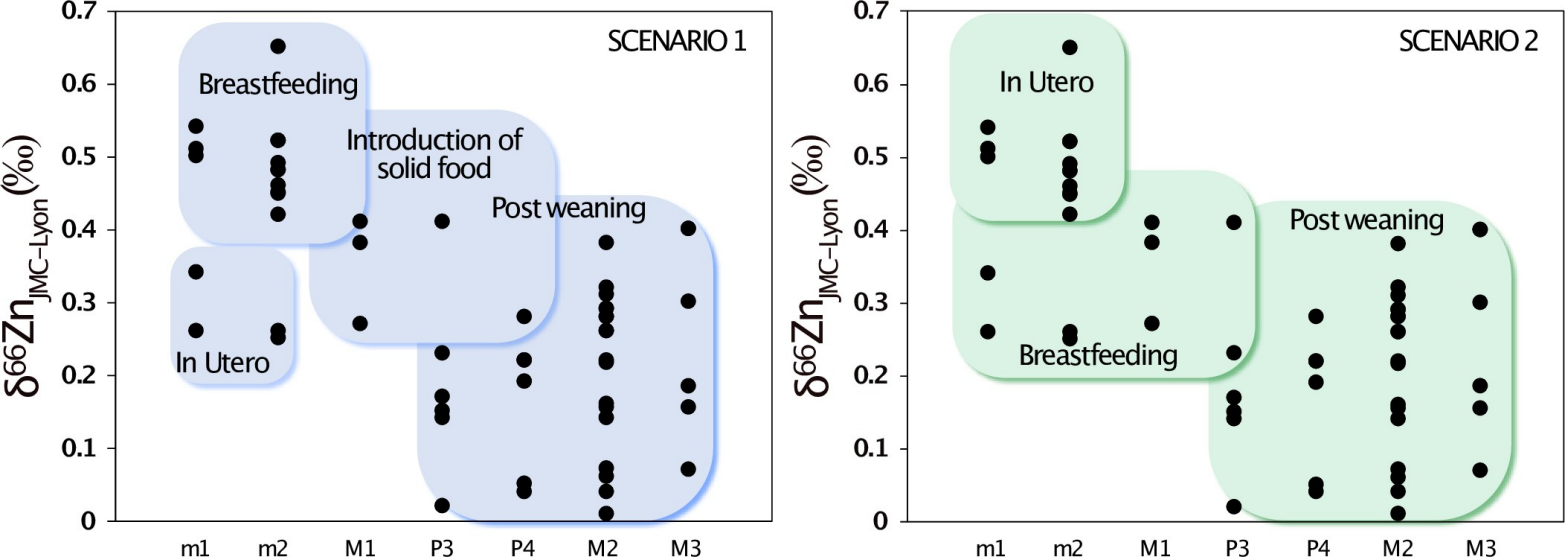
Possible scenarios for the evolution of enamel δ^66^Zn values in teeth during childhood.

Many of the teeth presented in this study have formed their enamel during dietary transitions: milk teeth often record *in utero* and breastfeeding signatures. In some cases, they can even record a third dietary transition: the introduction of solid food. The M1 enamel also records successively: exclusive breastfeeding, breastfeeding in combination with solid food consumption, and to a lesser extent towards the end of crown formation, post-weaning signatures. It therefore remains difficult to identify with certainty which δ^66^Zn values can be associated with dental enamel formed *in utero*, during the exclusive breastfeeding period or when solid food is present in the diet. From the trend seen in Fig 12, we can suggest the two scenarios (Fig 14) described below, in order to explain what causes Zn isotope variations between tooth types. **Scenario 1 (S1):** The lowest δ^66^Zn values observed in the deciduous m1 could correspond to *in utero* life, while the highest values observed in all studied deciduous m1 and m2 would result from exclusive breastfeeding. Indeed, all m2 show higher δ^66^Zn values than m1 after which they shortly started forming. The permanent M1, formed during the first three years after birth, would record a mixed signature of breastfeeding and solid food consumption. The P3, P4, M2 and M3 then record post-weaning δ^66^Zn values. **Scenario 2 (S2):** The dental enamel formed *in utero* records the highest ratios, while post-weaning formed enamel shows the lowest. The mother milk would then have intermediate Zn isotope ratios between blood (placental diet) and bulk diet (solid food). In both scenarios, the variation of δ^66^Zn values over time could also be due to a differential fractionation related to the maturation of the intestinal tract.

For the juvenile individual 63949 (Jacobin convent), the two deciduous m1 were sampled at the cusp tip of the crown to note that the sample of lower m1 includes some enamel from the middle part of the crown (S5 Table). This could explain the isotope difference observed between the two teeth, provided that the upper m1 sample exhibits a lower δ^66^Zn and contains more enamel formed *in utero* (Figs 11, 12 and 13). Moreover, the deciduous central incisors, which mainly form their enamel *in utero*, show the lowest Zn isotope ratio among all deciduous teeth. This would lend support to Scenario 1. The differences observed between the child and the adult at the Jacobin convent may first be related to the fact that the top of the crown (cuspal enamel) was often sampled for the child, while enamel was more frequently sampled in the middle of the crown (lateral enamel) for the adult. This means that the child´s samples contain material from earlier stages of enamel formation, while the adult´s samples reflect enamel from a time period when the individual was still breastfed. It may also be that the adult individual 20106 was weaned before the child 63949, a likely scenario considering their different socio-economical group (Supplementary Information).

Does Scenario 1 fit with what could be expected from the isotope composition of the food products and animal tissues? In animals, a pilot study showed that dairy products tend to have higher δ^66^Zn values than meat products [45]. However, those δ^66^Zn values are even higher in plant products. One could consider that a diet in which plants are the main source of proteins, as hypothesized for the Lapa do Santo population [9], should be associated to higher δ^66^Zn values [27,42]. Nevertheless, most of the Zn in the diet comes from animal products [46], and the adult diet of the individuals from Lapa do Santo did contain meat as attested by the abundant presence of burnt bones of small and middle-sized animals in the site [9]. Moreover, it is not entirely clear which was the dietary source of animal protein in the Lapa do Santo population. On the one hand, carbon and nitrogen isotopic data indicates a low position in the trophic chain. On the other hand, there were abundant middle- and small-sized burnt bones of mammals like rodents, deer, peccary and armadillo at the site, along with bone remains of reptiles and marsupials as well as abundant shell remains from terrestrial snails [9]. The δ^66^Zn values of non-mammalian tetrapods are still unknown [9]. In addition, the δ^66^Zn values of the fauna of Lapa do Santo have not been analyzed yet, so the comparison with other existing δ^66^Zn values on humans from other locations cannot be done. Possible site effects [7,47] could indeed induce variation in local Zn isotope compositions of the food webs. It is therefore possible that the post-weaning diet of the Lapa do Santo population is associated with lower δ^66^Zn values than the breastfeeding one. In Scenarios 1 and 2, we therefore hypothesized that mother milk is enriched in heavy Zn isotopes compared to the main sources of Zn in Lapa do Santo individuals’ diets. Considering the question of the differences between the enamel parts formed *in utero* or during the child’s infancy, Scenario 2 (S2) suggests that *in utero* δ^66^Zn values might be 0.3‰ above that of the permanent teeth, which would correspond, somehow, to one trophic level below the post-weaning diet [7,18,28]. This could be explained by the placental diet based on the nutrients coming from the mother’s blood, in a way similar to what happens with Ca isotopes [48]. Indeed, δ^66^Zn in human blood is enriched by 0.4‰ compared to human or animal muscles [49,50], which corresponds to the above mentioned difference between P4, M2 and M3 and the parts of m1 and m2 enamel assumed to be formed *in utero* (S2). However, based on the Jacobin convent isotope data, Scenario 1 would be the most likely. A progressive maturation of the intestinal tract may be associated to a differential fractionation of Zn overtime, in a similar way to what happens for Sr and Ca [51], which are not discriminated by children’s intestinal tracts. More work is needed to better understand the Zn isotopic differences between human milk and food products, as well as the associated fauna of the Lapa do Santo individuals to better constrain diet-related factors controlling intra-population and intra-individual variability of tooth enamel δ^66^Zn values. However, the similarity of the patterns observed in two very contrasting contexts (the Paleoamericans and hunter-gatherers of the tropical and prehistorical site of Lapa do Santo, Brazil and the medieval urban Bretons of the temperate site of the Jacobin convent, Rennes, France) is striking.

## 5. Conclusion

The release of Zn from different types of gloves into acidic solution confirms previous results and highlights the potential of Zn contamination during chemical sample treatment. Vinyl gloves showed the lowest contamination potential when quantifying Zn isotopic ratios, although they still release some Zn and are the less protective type of gloves for the experimenter against chemicals. A recommendation when working in the clean lab would be to wear two types of gloves, a pair of vinyl gloves (minimum contamination) superimposed on a pair of nitrile gloves (best protection for the user). Cleaning the gloves prior to utilization is not efficient because of the low amount of Zn released in water, and the safety risks that would be associated with an acid step. However, we did not detect any Zn contamination during enamel sampling, although the enamel making the outer surface of the tooth crown may have accidently been in contact with the nitrile or latex gloves. Thus, Zn is mostly released from gloves in the context of an acidic environment and not by mechanical contact with the tooth. Still, we would recommend not to wear any plastic gloves during tooth sampling and if protection is needed, we recommend wearing textile gloves instead. The enamel δ^66^Zn values of the Lapa do Santo humans display a trend where teeth formed during the earlier stages of life have the highest values, while teeth forming during later childhood show the lowest values. Enamel samples of deciduous teeth have the highest δ^66^Zn values, which could be best explained by elevated δ^66^Zn values of maternal blood and milk, enriched in heavier Zn isotopes compared to the adult diet of the Lapa do Santo humans, as reflected in the lower δ^66^Zn values of their permanent molars. Teeth mineralized during the two first years of life have intermediate enamel δ^66^Zn values, probably due to the mixed signature of the consumption of solid food and mother milk. Future work on the faunal δ^66^Zn values of this site will help to better interpret the diet of the different individuals of this population. More work is needed to explain the Zn isotope variations during childhood in this study, to further explore if Zn can be a promising proxy to trace age at weaning–similarly to Ca isotopes. Moreover, this work confirms the interest to use Zn isotopes in dental enamel for dietary reconstruction of archeological mammals (fauna and humans) in tropical settings [7], since bone and tooth collagen preservation in Lapa do Santo was poor, while enamel δ^66^Zn values seem pristine and a promising dietary tracer.

## Supporting information

S1 Fig Zn isotope composition in different teeth coming from the same Lapa do Santo individuals. Single enamel δ^66^Zn measurements per tooth and of each individual are connected by a line. Burial 23 contains 4 individuals (yellow), while burials 11, 15 and 16 involve only one individual (blue, green and red respectively).
(DOCX)

S2 Fig Three-dimensional model of the left permanent maxillary first molar (Specimen ID: SEVA 3598_3) of the Jacobins convent child showing the sampling strategy used for the Zn isotope analysis. A chunk of enamel and dentine is first detached from the crown using a pre-existing fracture (darker grey in the sectioned enamel). This piece of tooth is then cut in three sub-samples, from which the dentine will be removed to leave only the enamel to be used for the Zn analyses. The middle portion of the sample (natural colors) is preserved and was not used for analyses. The red subsample concerns the cuspal part of the crown and thus represents the earliest stages of enamel growth (until ~1.5 years), while the green subsample represents crown completion (~until 3 years of age).
(DOCX)

S3 Fig N isotopes obtained on the tooth roots of the child 63949 (Jacobins convent, Rennes). (DOCX)

S4 Fig Matching of the stress events observed in the dentine on the 3D model of the lower left M1 of the child of the Jacobins convent and a 200 μm-thick virtual 2D section (taken where is the red line on the 3D model). The turquoise arrows show the perfect alignment of the virtual 2D section and the 3D model. A strong stress precedes crown completion (pink arrows in enamel and dentine) and produce a marked enamel hypoplasia on the enamel outer surface. From crown completion (~3 years) to death (~6 years), the child has undergone strong and chronic stress events (green and orange).
(DOCX)

S5 Fig Relationship between Zn concentrations and isotope ratios in the teeth of the child and the adult from the Jacobins convent. (DOCX)

S1 Table Zn isotope ratios of the standards (measured and expected values). (XLSX)

S2 Table Zn isotope and concentration data for the glove tests. (XLSX)

S3 Table Zn isotope ratios and concentration from the Lapa do Santo teeth with anthropological data. Delta values are expressed in ‰, concentrations in ppm.
(XLSX)

S4 Table. Data used for the Linear Mixed Model (XLSX)

S5 Table Zn isotope data for the Jacobin convent teeth (individual 63049 and 20106). Delta values are expressed in ‰. The type of teeth sampled, the part sampled as well as the corresponding formation ages are also listed.
(XLSX)
