# Supplementary material for: Zinc isotope variations in archeological human teeth (Lapa do Santo, Brazil) reveal dietary transitions in childhood and no contamination from gloves
Source: PLoS One. 2020 May 14;15(5):e0232379. doi: 10.1371/journal.pone.0232379 (PMC7224499; doi:10.1371/journal.pone.0232379)
Supplement: S1 Fig — Single enamel δ66Zn measurements per tooth and of each individual are connected by a line. Burial 23 contains 4 individuals (yellow), while burials 11, 15 and 16 involve only one individual (blue, green and red respectively). (DOCX) [file pone.0232379.s001.docx]

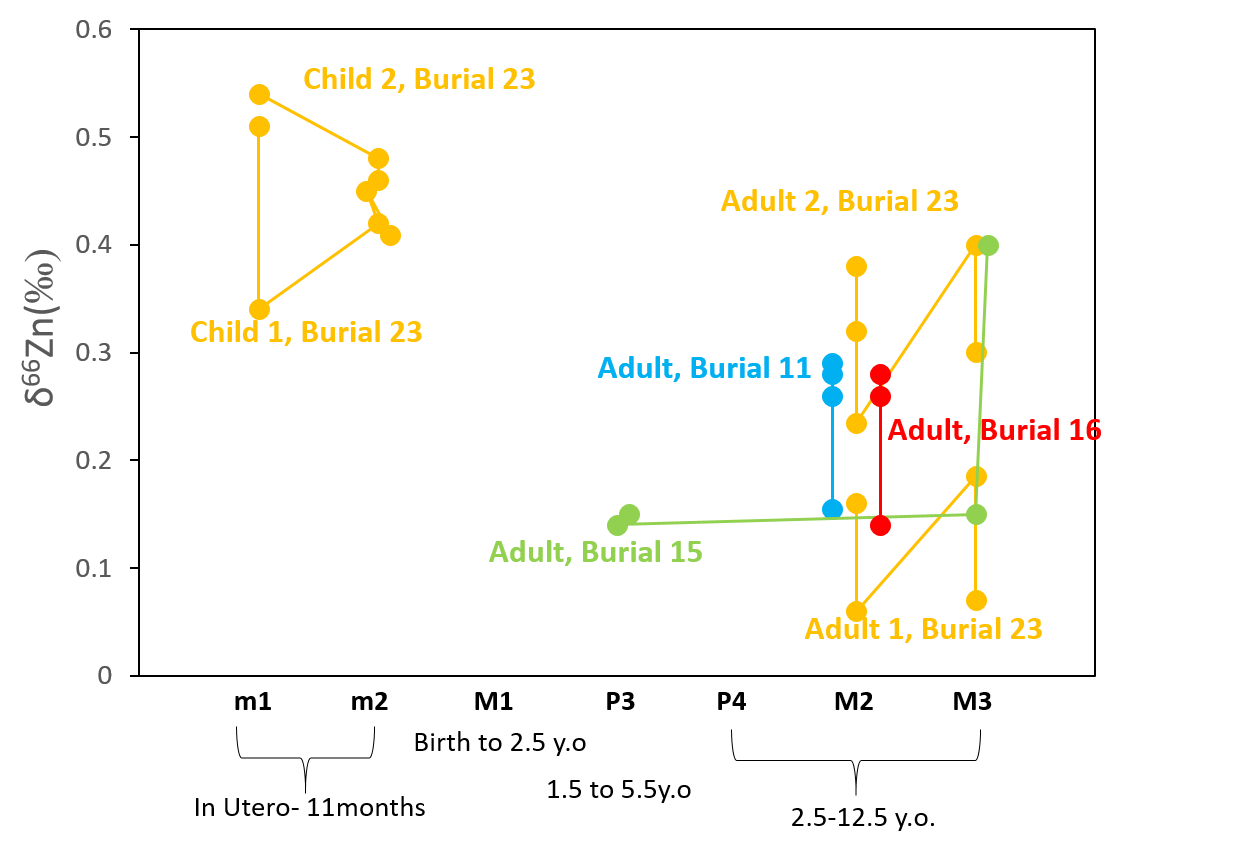


**Figure S1.** Zn isotope composition in different teeth coming from the same Lapa do Santo individuals. Single enamel δ^66^Zn measurements per tooth and of each individual are connected by a line. Burial 23 contains 4 individuals (yellow), while burials 11, 15 and 16 involve only one individual (blue, green and red respectively).
