# Supplementary material for: Zinc isotope variations in archeological human teeth (Lapa do Santo, Brazil) reveal dietary transitions in childhood and no contamination from gloves
Source: PLoS One. 2020 May 14;15(5):e0232379. doi: 10.1371/journal.pone.0232379 (PMC7224499; doi:10.1371/journal.pone.0232379)
Supplement: S2 Fig — A chunk of enamel and dentine is first detached from the crown using a pre-existing fracture (darker grey in the sectioned enamel). This piece of tooth is then cut in three sub-samples, from which the dentine will be removed to leave only the enamel to be used for the Zn analyses. The middle portion of the sample (natural colors) is preserved and was not used for analyses. The red subsample concerns the cuspal part of the crown and thus represents the earliest stages of enamel growth (until ~1.5 years), while the green subsample represents crown completion (~until 3 years of age). (DOCX) [file pone.0232379.s002.docx]

**
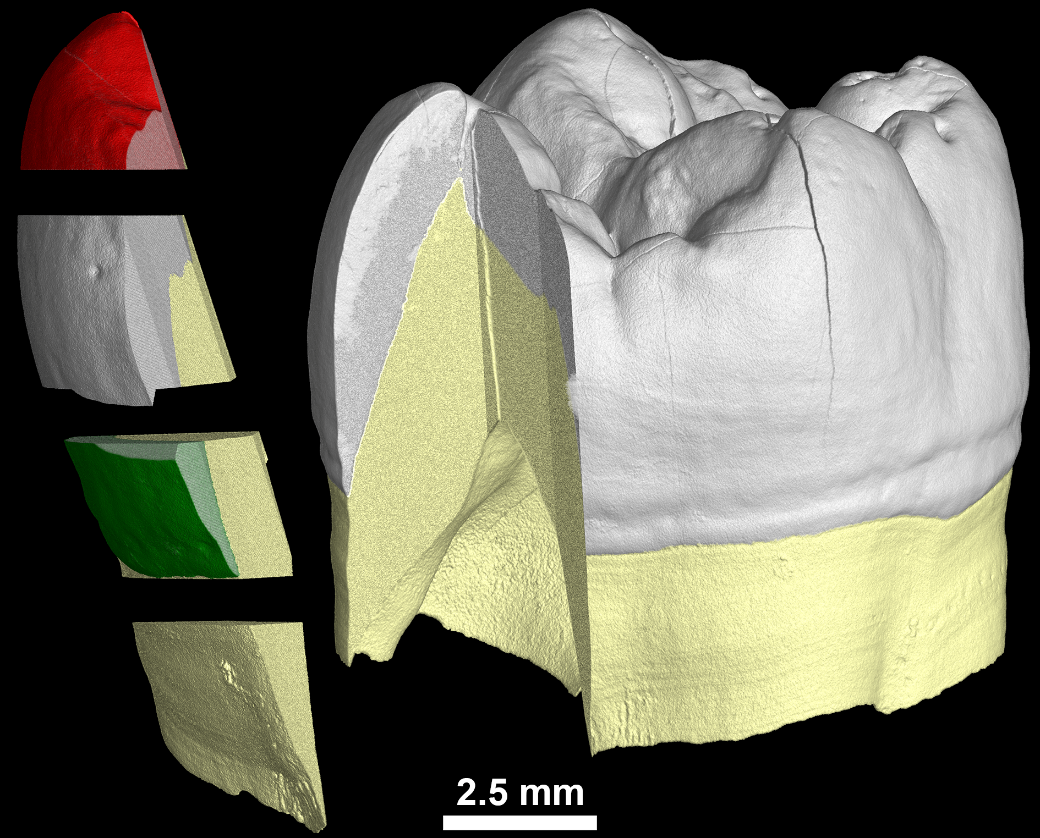
**

**Figure S2.** Three-dimensional model of the left permanent maxillary first molar (Specimen ID: SEVA 3598_3) of the Jacobins convent child showing the sampling strategy used for the Zn isotope analysis. A chunk of enamel and dentine is first detached from the crown using a pre-existing fracture (darker grey in the sectioned enamel). This piece of tooth is then cut in three sub-samples, from which the dentine will be removed to leave only the enamel to be used for the Zn analyses. The middle portion of the sample (natural colors) is preserved and was not used for analyses. The red subsample concerns the cuspal part of the crown and thus represents the earliest stages of enamel growth (until ~1.5 years), while the green subsample represents crown completion (~until 3 years of age).
