# Supplementary material for: Zinc isotope variations in archeological human teeth (Lapa do Santo, Brazil) reveal dietary transitions in childhood and no contamination from gloves
Source: PLoS One. 2020 May 14;15(5):e0232379. doi: 10.1371/journal.pone.0232379 (PMC7224499; doi:10.1371/journal.pone.0232379)
Supplement: S4 Fig — The turquoise arrows show the perfect alignment of the virtual 2D section and the 3D model. A strong stress precedes crown completion (pink arrows in enamel and dentine) and produce a marked enamel hypoplasia on the enamel outer surface. From crown completion (~3 years) to death (~6 years), the child has undergone strong and chronic stress events (green and orange). (DOCX) [file pone.0232379.s004.docx]

**
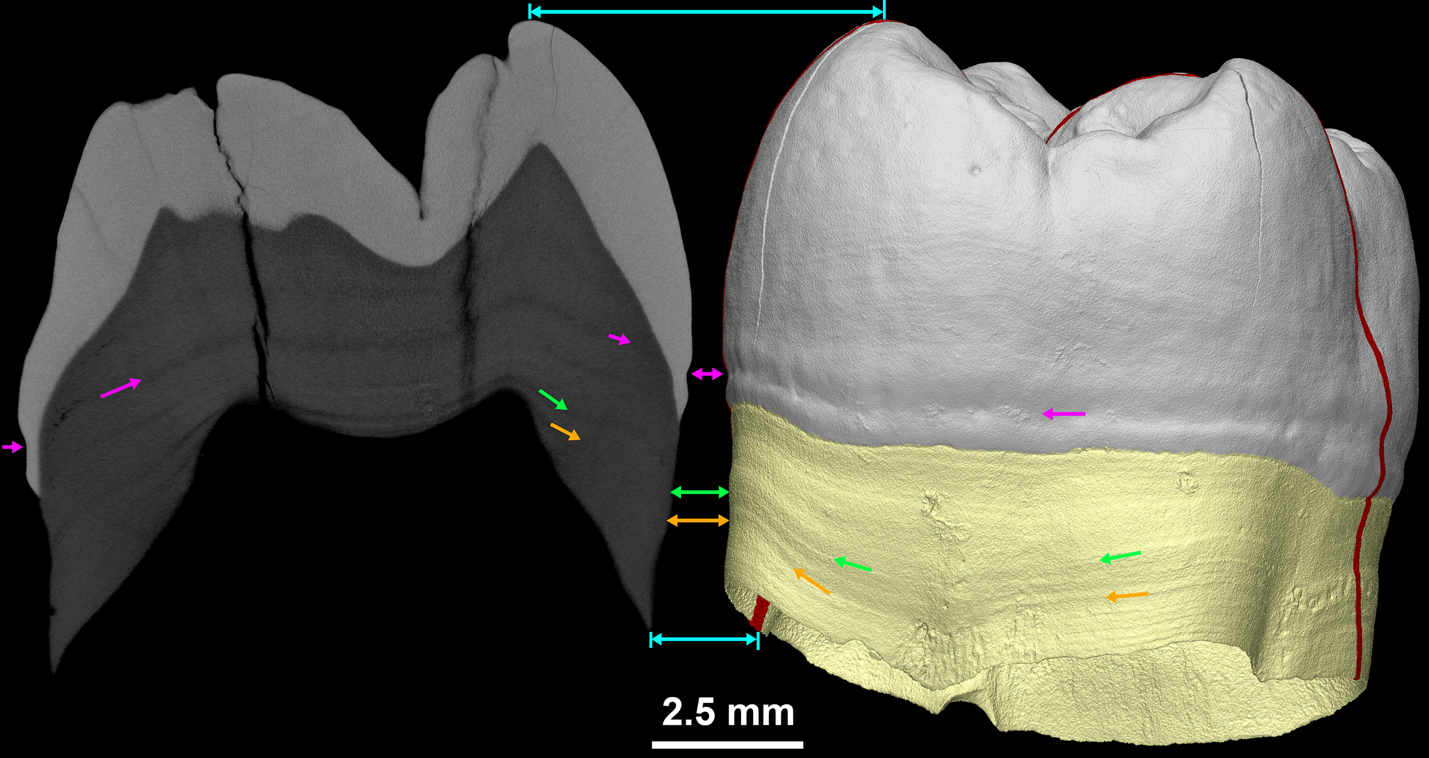
**

**Figure S4**. Matching of the stress events observed in the dentine on the 3D model of the lower left M1 and a 200 µm-thick virtual 2D section (taken where is the red line on the 3D model). The turquoise arrows show the perfect alignment of the virtual 2D section and the 3D model. A strong stress precedes crown completion (pink arrows in enamel and dentine) and produce a marked enamel hypoplasia on the enamel outer surface. From crown completion (~3 years) to death (~6 years), the child has undergone strong and chronic stress events (green and orange).
