# Supplementary material for: Zinc isotope variations in archeological human teeth (Lapa do Santo, Brazil) reveal dietary transitions in childhood and no contamination from gloves
Source: PLoS One. 2020 May 14;15(5):e0232379. doi: 10.1371/journal.pone.0232379 (PMC7224499; doi:10.1371/journal.pone.0232379)
Supplement: S5 Fig — (DOCX) [file pone.0232379.s005.docx]

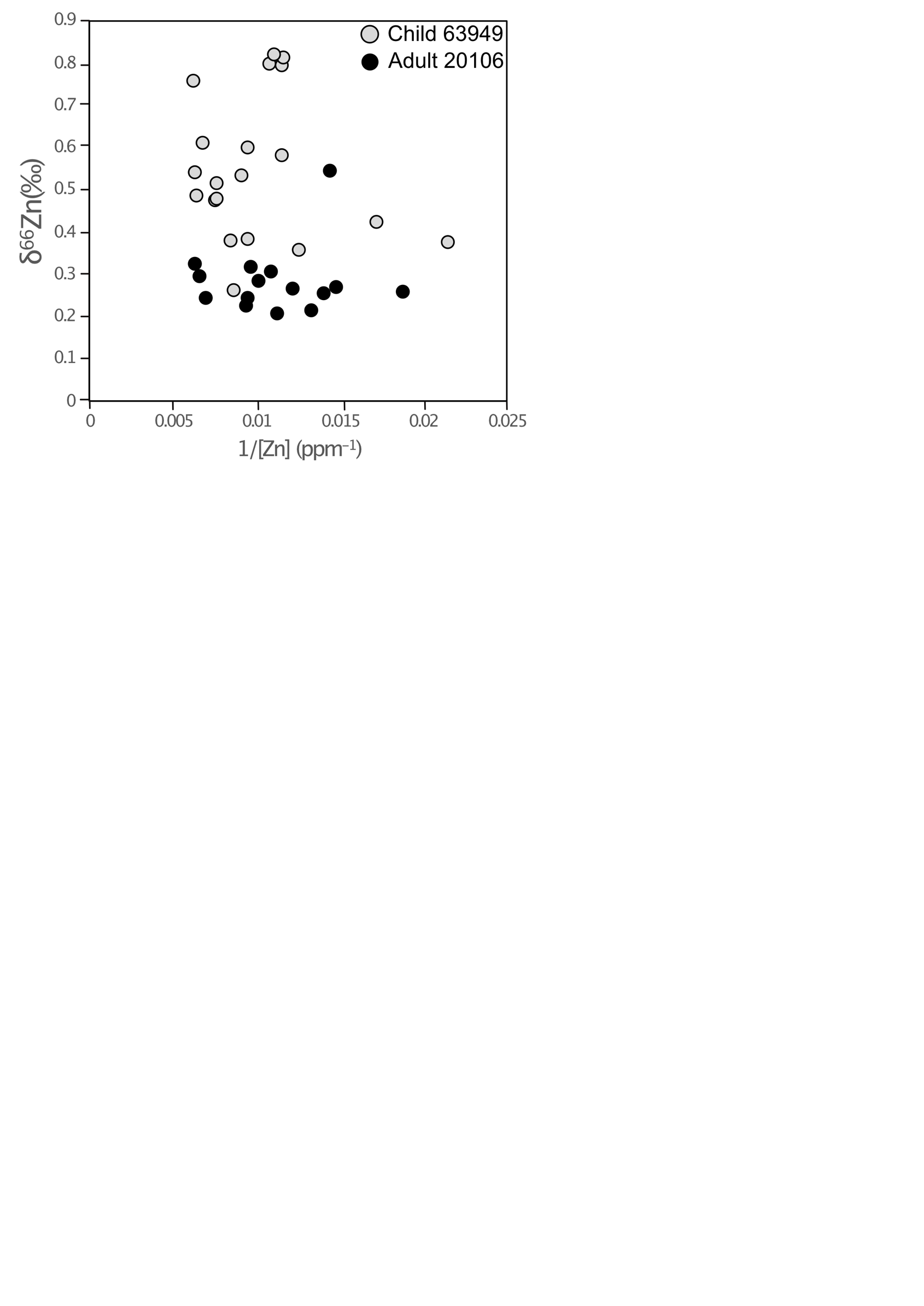


**Figure S5:** Relationship between Zn concentrations and isotope ratios in the teeth of the child and the adult from the Jacobins convent.
